# Regulation of p27^Kip1^ and p57^Kip2^ Functions by Natural Polyphenols

**DOI:** 10.3390/biom10091316

**Published:** 2020-09-13

**Authors:** Gian Luigi Russo, Emanuela Stampone, Carmen Cervellera, Adriana Borriello

**Affiliations:** 1 National Research Council, Institute of Food Sciences, 83100 Avellino, Italy; carmencervellera@libero.it; 2 Department of Precision Medicine, University of Campania “Luigi Vanvitelli”, 81031 Napoli, Italy; emanuela.stampone@unicampania.it

**Keywords:** p27^Kip1^, p57^Kip2^, polyphenols, EGCG, resveratrol

## Abstract

In numerous instances, the fate of a single cell not only represents its peculiar outcome but also contributes to the overall status of an organism. In turn, the cell division cycle and its control strongly influence cell destiny, playing a critical role in targeting it towards a specific phenotype. Several factors participate in the control of growth, and among them, p27^Kip1^ and p57^Kip2^, two proteins modulating various transitions of the cell cycle, appear to play key functions. In this review, the major features of p27 and p57 will be described, focusing, in particular, on their recently identified roles not directly correlated with cell cycle modulation. Then, their possible roles as molecular effectors of polyphenols’ activities will be discussed. Polyphenols represent a large family of natural bioactive molecules that have been demonstrated to exhibit promising protective activities against several human diseases. Their use has also been proposed in association with classical therapies for improving their clinical effects and for diminishing their negative side activities. The importance of p27^Kip1^ and p57^Kip2^ in polyphenols’ cellular effects will be discussed with the aim of identifying novel therapeutic strategies for the treatment of important human diseases, such as cancers, characterized by an altered control of growth.

## 1. Introduction

During the last two decades, polyphenols have gained huge interest as potential adjuvant cancer drugs and cancer-preventing molecules. In vitro studies have underlined that the beneficial proprieties of polyphenols rely in part on their antioxidant activity [1,2] and in part on their ability to modulate signaling pathways related to cell cycle progression and cell survival [3,4]. The convergence points of these cellular processes are represented by cyclin-dependent kinase (CDK)s with their modulatory partners, cyclins, and CDK inhibitors (CDKIs). Particularly, CDKI proteins play fundamental roles in tuning the activity of CDK complexes in response to endogenous and exogenous stimuli, including drug treatments. Regarding CDKI classification, two families are recognized: p16^INK4a^, p15^INK4b^, p18^INK4c^, and p19^INK4d^ belong to the INK4 (INhibitors of CDK4(6)) family, while p21^Cip1^, p27^Kip1^, and p57^Kip2^ (hereinafter p21, p27, and p57) are members of the CIP (CDK Interacting Proteins)/KIP (Kinase Inhibitory Proteins) family [5].

For a long time, CIP/Kip protein functions have been mainly related to their ability to bind and inhibit different cyclin–CDK targets [6,7,8]. As a matter of fact, these proteins restrain growth during (i) development, (ii) differentiation, and (iii) responses to various cellular stresses and drug treatments. It is important to underline that, although apparently redundant, each CIP/Kip member cannot be completely surrogated by its siblings, suggesting specific roles [9]. p21 is mainly, but not only, a p53 effector and is upregulated when p53 is activated, as is the case, for example, in response to different DNA-damaging conditions [10]. This CDKI mediates cell cycle arrest in the G1 and G2 phases to allow DNA repair or to induce apoptosis when the damage cannot be repaired. Alternatively, p53-independent signaling, cell growth factors (such as PDGF, FGF, and EGF), or epigenetic modulators have been reported to be capable of activating p21 expression [11]. p27 has been mainly considered as a major conduit of specific external stimuli to control the decision of the cell to escape or enter the cell cycle [12]. Finally, studies on p57 have underlined the importance of this CDKI in the cell cycle control during embryonic development and maturation [13]. Accordingly, its tissue expression shows a restricted pattern during organogenesis and an even more restricted one in adult life [14].

However, as always happens in nature, the truth of things appears to be much more complex. Continuously, pivotal new functions have been ascribed to all CDKIs, several of them being CDK-unrelated. Today, CIP/Kip proteins have been associated with the control of multiple complex processes, such as cell proliferation, apoptosis, autophagy, survival, the DNA-damage response, gene transcription and translation, cytoskeleton rearrangement and dynamics, mitotic steps, and many other functions [9,15,16,17].

## 2. p27 and p57 Proteins’ Major Features, Properties, and Functions

**Figure 1:**
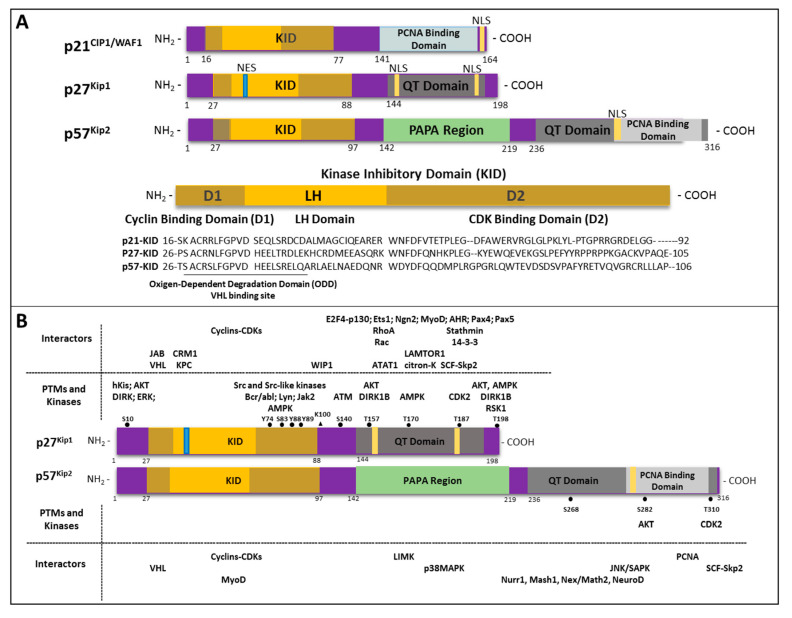
Domain structures of cyclin-dependent kinase (CDK) Interacting Protein (CIP)/Kinase Inhibitory Protein (Kip) proteins with the main post-translational modifications (PTMs), putative kinases, and interactors of p27Kip1 and p57Kip2. Panel (**A**) Schematic representation of p21Cip1, p27Kip1, and p57Kip2 protein domain structures. The three siblings share a highly conserved N-terminal domain called the Kinase Inhibitory Domain (KID) that includes the cyclin-binding domain (D1) and the CDK-binding domain (D2) connected by a linker helix (LH). The Oxygen-Dependent Degradation Domain (ODD), reported for the binding of Von Hippel Lidau (VHL), partially overlaps with the D1 domain. NES (Nuclear Export Signal); NLS (Nuclear Localization Signal). Panel (**B**) Known PTMs, kinases, and interactors of p27 and p57. In the panel, the domain structures of p27 and p57 are reported. The main sites of phosphorylation for both the proteins are indicated with spots along the sequence. At the site of the phosphorylation, the putative kinases responsible for the phosphorylations are also indicated. One acetylated site is reported for p27 (K100), and it is indicated on the protein with a triangle. The interactors of the two proteins, mentioned and described in the text, are also reported in correspondence with their binding site.

p27 and p57 (also known as p21) share a certain degree of homology in their structure, particularly in their N-terminal regions, where a conserved domain is allocated, necessary, and sufficient for binding and inhibiting CDK complexes. As shown in Figure 1A, this protein region, defined as the Kinase Inhibiting Domain (KID), consists of a cyclin-binding subdomain (termed D1) and a CDK-binding subdomain (termed D2) joined by a linker subdomain of about 22 residues (called LH). Recently, by means of in silico studies, an ODD-like motif (Oxygen-Dependent Degradation-like motif) has been recognized in their N-terminal domains with a putative interaction with the VHL (Von Hippel Lindau) protein, which, however, needs further investigation [18].

Differently, the C-terminal domains (CTDs) of the two proteins vary in length and sequence, exhibiting either similar or different features. Both CTDs show putative nuclear localization signals. Moreover, the p27 and p57 C-terminals contain a QT domain made up of glutamine and threonine repeats involved in protein–protein interactions [19]. Inside the QT domain, p57 displays a Proliferating Cell Nuclear Antigen (PCNA)-binding domain, which is also present in the C-terminal of p21. In addition, it has been reported that the QT box of p57 directly binds the kinase JNK/SAPK (c-Jun NH2-terminal kinase/stress-activated protein kinase), determining its inhibition [20,21]. p21 is also able to bind and suppress the activity of JNK/SAPK, although via its amino-terminal domain. On the contrary, p27, which contains a QT domain, does not inhibit JNK/SAPK activity. An additional unique domain (made up of PAPA, proline–alanine, repeats) is also present in the central portion of p57, upstream of the QT domain, whose functions have not been completely elucidated, although it confers peculiar biochemical and biophysical properties to the protein [22].

p27 and p57 are intrinsically disordered proteins (IDPs), having a low degree of stable tertiary structures, which can be adopted, totally or in part, upon binding to specific interactors [23,24]. This property, strongly diverging from the classic structure–function paradigm, confers high conformational flexibility to these proteins along with the ability to recognize, and interact with, a plethora of different partners. In turn, the large plasticity gives to IDPs the possibility of exerting a multiplicity of functions. On the other hand, in view of their complex roles, the disruption of p27/p57 homeostasis is associated with numerous diseases, including cancer and neurodegeneration [25,26].

### 2.1. p27: A Key Ambiguous Protein with Multiple Contrasting Roles

#### 2.1.1. p27 Structure and Function

p27 was first identified in 1994 as a CDKI homolog of p21 [27,28,29]. Since then, myriad papers have reported p27 levels to increase in response to anti-mitogenic molecules, differentiation signals, cell-to-cell contact, mitogen starvation, loss of adhesion to the extracellular matrix, and other conditions. Although able to bind and inhibit virtually all cyclin–CDK complexes, p27 certainly seems to inhibit cyclin E(A)/CDK2, particularly at the G1/S boundary of the cell cycle, while some degree of ambiguity exists regarding its activity on cyclin D–CDK4/6 [30]. Specifically, the kinase is inhibited in quiescent cells, while in cycling cells, p27 participates in the assembly, nuclear import, and allosteric activation of the cyclin D–CDK4/6 enzymatic complex [31,32]. The activity of p27 on cyclin B/CDK1 complexes was first evoked by the observation that p27 inhibits the kinase in CDK2-ablated mice. Accordingly, CDK1 activity upregulation has been reported in p27^-/-^ animals [33,34].

*Cdkn1b^−/−^* mice (*Cdkn1b* is the mouse p27-coding gene) show increased body size, hyperplasia of different organs, retinal dysplasia, neocortex alterations, and female sterility [35,36]. Moreover, heterozygous *Cdkn1b^+/−^* mice show a major susceptibility to developing tumors after chemical carcinogen treatment or irradiation, compared to their littermates, and spontaneously develop pituitary malignancies late in life [37,38].

Besides CDK-regulative activity, several other canonical and non-canonical functions, not all strictly CDK-dependent [17], have been demonstrated. This is certainly due to the disordered nature of p27 [8]. Furthermore, like for other IDPs, post-translational modifications (PTMs), predominantly phosphorylations, contribute to guiding the protein towards specific conformations, affecting stability, subcellular localization, and functions. A number of p27 phosphorylation sites have been reported. Only in limited cases have all the involved kinases and their specific biological roles been clearly identified [39,40]. The most characterized site of modification is Threonine 187 (T187), which is an in vivo and in vitro target of active CDK2- or CDK1-containing complexes [41,42]. Specifically, T187 phosphorylation induces the formation of a phosphodegron, essential for the recognition of and binding to S-phase kinase-associated protein 2 (Skp2), which represents the substrate-recognition subunit of the E3 ubiquitin ligase complex SCF–Skp2 (SCF, Skp-Cullin-F-box). The Skp2-mediated ubiquitination of p27 also requires Cks1 (CDK subunit 1), which cooperates as a linker protein in the binding between Skp2 and pT187-p27 [43]. The process finally guides p27 to proteasomal degradation inside the nucleus during the S–G2 phases, allowing cell cycle progression [41,44].

The phosphorylation of tyrosine(s) (Y74, 88, and 89) has been reported as being due to non-receptor tyrosine kinases, including Src and Src-like kinases, such as Lyn and Bcr/Abl. The main role of p27 tyrosine phosphorylation is to shift the role of p27 (when bound in the CDK2 catalytic cleft) from kinase inhibitor to CDK substrate, allowing T187 phosphorylation and consequent protein degradation. This pathway therefore removes the p27-dependent inhibition of CDK2 and favors cell cycle progression in response to mitogenic signals [45,46,47,48].

Serine 10 (S10) phosphorylation represents a quantitatively important p27 PTM. It has been reported to not only stabilize the protein in the nuclear compartment [49,50,51] but also to allow p27 exit from the nucleus into the cytosol when quiescent cells re-enter the cell cycle from the G0 phase [52]. The kinases reported as able to phosphorylate p27 in S10 comprise hKis, Dirk, ERK, AKT, and CDK5 (reviewed in [17,39]). In post-mitotic neurons, CDK5-dependent S10 phosphorylation stabilizes the p27 protein in the cytoplasm, particularly in the perinuclear region, where p27 is, in turn, able to inhibit the RhoA–ROCK pathway, causing the activation of cofilin, an F-actin-severing protein. This mechanism results in actin reorganization and neuronal migration [53].

The phosphorylation of threonine (T) 157 and/or 198 has been associated with p27 sequestration in the cytosol. As a matter of fact, T157, although not conserved in mouse p27, falls within the nuclear localization sequence in human p27 [54,55,56]. Reported roles for T198 phosphorylation include the assembly of cyclin D–CDK4/6 complexes, RhoA–ROCK pathway inhibition upon RhoA binding, stathmin interaction, and, therefore, both actin and microtubule cytoskeleton modification ([17,57] and references therein). The kinases identified as responsible for such PTM include AKT; RSK1, which is a downstream effector of PI_3_K and MAPK; and AMPK (AMP-activated protein kinase). Particularly, AMPK-dependent T198 phosphorylation has been associated with the activation of the autophagy process in response to nutrient-limited conditions [58]. Other sites of modification include S140, which is a target of ATM kinase involved in the early response to DNA damage [59]. The major sites of modifications of p27 and the putatively involved kinases are highlighted in Figure 1B, together with the major p27 interactors.

In summary, as evident from the numerous phosphorylated residues, p27 is a crucial protein on which multiple pathways converge, playing different functions both in the nucleus and in the cytoplasm [17].

In the nucleus, beside its cyclin–CDK inhibition activity, p27 participates in the control of pre-replicative and replicative complex formation and in the regulation of gene expression, due to the ability to interact with specific transcription factors on defined chromatin regions. Interactions have been reported with Ngn2, MyoD, AHR, Pax4, and Pax5 ([60] and references therein). It also binds the acetyltransferase and transcriptional co-activator PCAF, which, in turn, acetylates p27 on K100, modulating the stability of the protein [61]. ChIP-on-chip experiments performed in quiescent NIH373 cells and in mouse embryonic fibroblasts (MEFs) demonstrated that p27 significantly associates with E2F4–p130 complexes and with ETS1 and mainly acts to favor the recruitment of co-repressors such as HDAC1 and mSIN3A to the promoters of several target genes [62]. Other reports also demonstrated p27 C-terminal domain association with different intergenic chromatin regions, while the KID in the N-terminal domain favors the recruitment to chromatin of specific CDKs, causing, in some cases, their inhibition. However, the transcriptional effects of p27 appear to be only partially CDK-dependent, since the expression of p27CK^-^ mutants (which do not inhibit CDK activities) are still able to modulate the expression of certain target genes. p27 transcriptional regulation is also involved in embryonic stem cell differentiation and in the transcriptional repression of SOX2 [63], one of the three Yamanaka factors (Oct4, Klf4, and Sox2) involved in reprogramming pluripotent stem cells (iPSCs) [64]. On the other hand, a role of p27 as a pro-oncogenic transcriptional co-regulator of c-Jun in specific settings of cancer cells with hyper-activated PI_3_K/AKT has been recently reported [65].

In the nucleus, p27 may also play a role in the DNA-damage response (DDR). p27 involvement in the maintenance of genome integrity has been observed due to the phenotypical response of p27-null and heterozygous mice to chemical carcinogens or γ-irradiation. p27 may act at two different time points in response to DNA double-stranded breaks: in a first initiation phase, p27 is directly phosphorylated by ATM in S140, and this event might extend its half-life, allowing p27 to mediate the G1 checkpoint arrest; in a second phase, in conditions of persistent exposure to the DNA-damaging agent, p27 might be stabilized by a pathway independent of ATM/ATR activities, relying instead on p38MAPK activation [59]. In turn, through its CDK-inhibitory activity, p27 participates in a G2/M checkpoint arrest [66]. As reported recently, the phosphorylation of S140 is a target of WIP1 (Wildtype p53-Induced Phosphatase-1), whose overexpression and/or mutation have often been associated with oncogenesis [67].

In the cytosol, p27 has been associated with the control of programmed cell death or autophagy, and the modulation of both actin filaments and microtubule (MT) cytoskeleton dynamics, thus impacting growth, survival, cell movement, invasiveness, and metastasis [17]. Several pieces of evidence contribute to clarifying the role of p27 in the homeostasis of filaments of actin and in the correct organization of the contractile ring during cytokinesis. This is achieved through Rac, RhoA, and citron kinase involvement [68,69]. p27 is also able to interact with stathmin, an MT-destabilizing factor [70], and PRC1, a protein involved in microtubule cross-linking and central spindle formation, thereby affecting cell shape and motility and proper mitotic division [71]. p27-dependent augmented microtubule stability has also been linked to the control of cell cycle entry in mitogen-stimulated cells, since it favors the endocytic trafficking of H-Ras and its ubiquitination, causing the reduction of H-Ras–MAPK signaling [72].

Recently, the Nguyen group also discovered, in mice cortical neurons, the involvement of p27 in the stabilization of alpha-tubulin acetyl transferase 1 (alpha-TAT1), the principal enzyme involved in MT post-translational modifications [73], adding important pieces of information to the comprehension of p27′s activity on MT cytoskeleton dynamics in vesicle trafficking and neuron migration. In addition, the cytoplasmic CDKI is involved in the enhancement of epithelial–mesenchymal transition, a well-known pro-carcinogenic process, by inducing Twist1 upregulation via the activation of STAT3 [74]. Cytoplasmic p27 might promote invadopodia turnover and the formation of invadosomes, i.e., the active cellular structures playing a fundamental role in the degradation and invasion of the extracellular matrix. Mechanistically, it binds cortactin and facilitates the interaction with (and the phosphorylation by) PAK1, a Rac/CDC42-dependent kinase crucial for cytoskeleton reorganization [75]. Overall, p27 levels and localization impact cell shape and motility, either facilitating invasion and metastasis or, alternatively, exerting anti-migratory activities, depending on the cellular context.

p27 has been identified as a pivotal player during metabolic stress. Accordingly, p27 is involved in pathways correlated with autophagy and apoptosis [58]. Under conditions of cellular stress, p27 might thwart apoptosis by its capability of preventing Cdk2 activation as well as reducing the activity of Bax, a pivotal pro-apoptotic factor [76,77]. Other reports point to the pro-apoptotic effects of p27 [78]. Particularly, it has been shown that p27 overexpression in lung cancer cell lines induces cell cycle arrest and apoptosis through pRb expression downregulation [79]. Furthermore, in a cohort of patients with oral and oropharyngeal squamous cell carcinomas, the induction of spontaneous apoptosis was higher in cancers expressing p27 than in p27-negative tumors [80], due to the positive correlation with Bax expression.

In 2007, Liang and colleagues showed that the energy/nutrient-sensing kinase LKB1-activated AMPK phosphorylates p27 on S83, T170, and T198, increasing the protein stability and cytosolic localization of p27, with the consequent activation of autophagy and reduction of apoptosis [58]. p27 was also required for starvation-induced autophagy in MEFs, underscoring a function of p27 under conditions of scarcity of nutrients. Conversely, when the mTOR–raptor complex—a key serine/threonine kinase that stimulates protein synthesis, inhibiting autophagy—is active, it enhances p27 phosphorylation on T157 through the activation of SGK1 (Serum and Glucocorticoid-Inducible Kinase 1) and, in turn, favors its sequestration in the cytoplasm, causing cell cycle progression [81]. The importance of T198 phosphorylation, instead, was confirmed by transfection experiments with a phosphomimetic T198D-p27 mutant showing cytosolic protein localization and autophagy activation [58,82]. Finally, the silencing of *CDKN1B* affects autophagy caused by serum starvation or glucose withdrawal and induces apoptosis [58].

The mechanistic relationship between p27 and autophagy remains undefined but cannot be considered a function independent of CDKI activity, since CDK2/CDK4 depletion partially reproduces the effects of p27 on autophagy. Recently, a new mechanism has been proposed. Specifically, under amino acid deprivation, a p27 fraction localizes on lysosomes, where it binds LAMTOR1 and, in turn, hampers its capability of activating mTORC1. By this mechanism, p27 might induce autophagy. This finding is confirmed by experiments in p27^−/−^ MEFs that, when deprived of amino acids, show autophagy resistance. In this specific genetic setting and condition, autophagy activation was further inhibited through the sequestration in the cytosol of the transcription factor TFEB, which controls the expression of genes involved in lysosomal biogenesis and autophagy [83].

**Figure 2:**
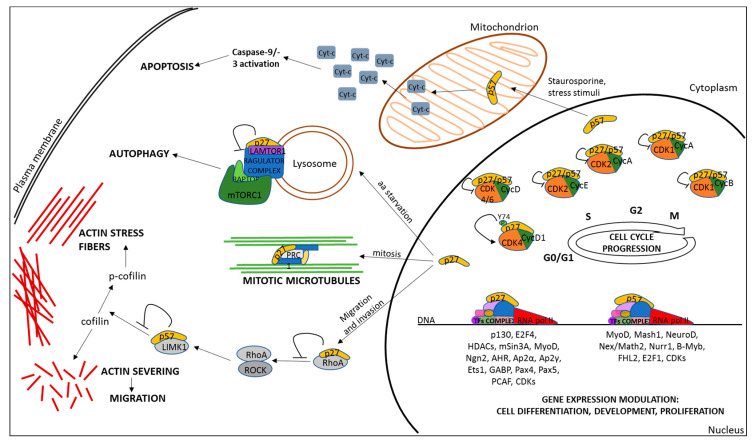
Schematic representation of the main roles of p27^Kip1^ and p57^Kip2^. In the nucleus, both the proteins are key modulators of the cyclin/CDK complexes, mainly inhibiting their activities and preventing cell cycle progression. In the case of phosphorylation on Tyrosine 74 of p27, this protein turns into an activator of the complex CDK4/cyclin D1, promoting cell cycle progression. In addition, p27 and p57 can take part in transcription factor complexes to promote or to inhibit gene expression during development, cell differentiation, and cell cycle progression. The main interactors are reported in correspondence with each protein complex on DNA. In the cytoplasm, the proteins can be involved in the regulation of several processes such as apoptosis, responses to several stressors, and cytoskeletal dynamics during mitosis, tumor cell invasion, and metastasis. aa (amino acids). Cyt. c (Cytochrome c). Refer to Section 2.1.1 and Section 2.2.1 for a detailed description of the activities of p27 and p57 represented in this figure.

In conclusion, the complex and heterogeneous phenotypic effects associated with p27 (schematically recapitulated in Figure 2) are often so divergent that they have stimulated the evocative definition, at least in the context of carcinogenesis, of a “Janus” protein with a dual role, i.e., tumor suppressor or tumor promoter. However, based on the continuously identified novel functions of p27, a definition of “many-faced protein” appears more appropriate. This high heterogeneity is probably dependent on two major factors, i.e., its structure and the specific context (cell phenotype, environmental conditions, interacting protein abundance, and cell treatments) in which the protein works.

#### 2.1.2. Mechanisms of p27′s Cellular-Level Regulation

The mechanisms controlling cellular p27 levels are multiple, namely, the regulation of *CDKN1B* transcription, the control of mRNA translation efficiency, and protein-targeted degradation. To date, the major known activators of *CDKN1B* transcription are members of the FoxO (Forkhead box class O) family of transcription factors [84]. Thus, all the pathways that modulate FoxO activity, including PI_3_K/AKT, control p27 contents [85,86]. Intriguingly, factors activating AKT comprise various cytokines and the cAMP/PKA pathway. FoxO proteins also promote p27 nuclear localization and reduce the levels of COP9 subunit 5, a protein involved in p27 degradation [46]. Other factors positively affecting p27 transcription include, among others, Sp1, E2F-1, BRCA1, Kruppel-like factor 7, and nuclear hormone receptors. Transcription factors that inactivate the p27 promoter comprise c-Myc, Id3, Hes1, Notch/HTRT1, and HDACs [87].

The 5′-untraslated region (UTR) of the p27 mRNA has been described as highly structured. It includes an IRES (Internal Ribosome Entry Sequence) and an upstream ORF (uORF) sequence located in a cell cycle regulatory element (CCRE). The IRES probably sustains the translation under conditions of unfavorable growth conditions [88]. The CCRE is formed by a C/G rich sequence and a uORF that, analogously to other uORFs, might code for a peptide regulating the main p27-coding ORF translation, probably during cell division cycle progression. p27 translation is also regulated by different miRNAs (mainly miR221 and miR222), although the precise details of this mechanism are not completely understood [89].

As anticipated before, the levels of p27 are controlled mostly by proteasomal degradation. Different mechanisms of p27 proteolysis have been described that occur in different phases of cell cycle division. G1–S transition requires cytosolic degradation by a ubiquitination mechanism that, apparently, does not require an initial phosphodegron formation. Specifically, it is driven by KIP1 Ubiquitylation Promoting Complex (KPC). On the other hand, the shuttling of p27 from the nucleus to cytosol appears to be the initial targeting step for KIP1-dependent degradation and seems to involve the phosphorylation of S10 and binding to CRM1 [90]. Conversely, during the S/G2 phases, p27 removal occurs mostly in the nuclear compartment by means of the above-described process, which involves the sequential phosphorylation of T187, Skp2-dependent ubiquitination, and proteasomal degradation [41]. Other reported mechanisms of p27 catabolism require the activity of the protease calpain, as shown by [91] in a model of preadipocyte mitotic clonal expansion.

### 2.2. p57, an Unrevealed Protein Involved in Cell Cycle Control, Cell Differentiation, Cell Death, and Senescence

#### 2.2.1. p57 Structure and Function

Among the CIP/Kip family proteins, p57 is certainly a less-characterized member. This is probably due to its scarce expression in adult organisms, restricted to a few tissues [14,22,92]. Conversely, it is largely present in embryonal tissues, suggesting a central role in differentiation and morphogenesis. Accordingly, *Cdkn1c*-null mice (*Cdkn1c* and *CDKN1C* are the mouse and human genes encoding p57, respectively) are not vital or die soon after birth. The modified animals (or embryos) present developmental defects that partially mimic the human Beckwith–Wiedemann Syndrome phenotype, a rare genetic disease characterized by developmental defects, overgrowth, and tumor predisposition. On the contrary, the excess of p57 protein in mice leads to an increase in embryonic lethality and a reduction in the size of the body, suggesting that the p57 dosage needs to be finely tuned to ensure correct morphogenesis [13,93]. Furthermore, the substitution of *CDKN1C* with *CDKN1B* (in other words, the introduction of the *CDKN1B* gene at the *CDKN1C* locus, leaving the transcriptional control mechanism intact) cannot completely compensate the role of p57, suggesting peculiar functions of the protein [94]. Various reports suggest p57 involvement during the quiescence and maintenance of adult stem cells. To date, this has been clearly proved in hematopoietic and neuronal stem cells [95,96]. The protein also increases during the differentiation of skeletal muscle myoblasts, podocytes, keratinocytes, and cortical precursors [97,98].

Additional features clearly differentiate p57 from its siblings. First, the genomic localization of *CDKN1C* (chromosome 11p15.5) is of particular relevance, in that this region is highly imprinted and only the maternal allele is expressed. As a consequence, mutations that affect the maternal allele behave as in homozygosity. This finding is certainly important in explaining the phenotypes associated with *CDKN1C* genetic alterations. A second matter of complexity is that, even more than for p27, an extended part of the protein has an unfolded status with huge variability, especially in length, among species [99]. Third, as for p27, PTMs might influence the fate of the protein. To date, little information on p57 PTMs is available. Phosphorylation has been reported for residues S268, S282, and T310 (Figure 1B). On the other hand, with the protein being an IDP, a clear characterization of its PTMs is mandatory. The most frequently confirmed site of phosphorylation is T310. Phosphorylation on T310 plays a role analogous to that of that on p27 T187 and is involved in a ubiquitin-proteasome-dependent mechanism of degradation [100]. In brief, phosphorylation on T310 determines the formation of a phosphodegron, which functions as a recognition site for the Skp2 protein of the E3 ubiquitin ligase SCF complex.

Upon analyzing the domains of the protein (Figure 1), it can be observed that the amino-terminal region of p57 is not only necessary for the binding of cyclin–CDK complexes through the KID domain, as described for p27, but is also involved in numerous interactions during differentiation processes. It can interact with helix–loop–helix transcription factors, such as NeuroD, Nex/Math2, and Mash1 for neuronal differentiation [101], and Nurr1, an orphan nuclear receptor, particularly important for dopaminergic neuron differentiation [102]. p57 also binds B-Myb, a transcription factor playing a key role during early embryonic development [103]. Several studies have examined the effect of p57 on the stability of MyoD, one of the muscle-specific transcription factors expressed in proliferating myoblasts prior to terminal differentiation [104]. The authors reported two different mechanisms, namely, the inhibition of cyclin E/CDK2 kinase activity and a physical interaction with MyoD, thus promoting MyoD–DNA binding during the myogenic differentiation [105]. Of interest, recently, it has reported that p57 is able to interact with VHL, starting from the consideration that, like the other CIP/Kip proteins, it shows an ODD-like motif partially overlapping the KID in the N-terminal domain. Even though the functional meaning of the interaction needs to be elucidated, a regulatory connection between the HIF-1α and p53 pathways and the control of the cell cycle is proposed [18].

The central part of the protein is a characteristic that distinguishes p57 from p21 and p27. Notably, human p57 is a 316-amino-acid protein with a calculated MW of 32,177 but migrates at 57 kDa in SDS-PAGE electrophoresis, hence the name p57 (this behavior is not observed for p21 and observed only to a minor extent for p27). An explanation may lie in the presence of the PAPA region, a type of hinge made up of proline and alanine repeats between the N- and the C-ends of the protein, whose function is still unclear. A few reports suggest that it is important for the modulation of protein–protein interactions. Particularly, this region was reported to be involved in the binding of p57 with LIMK1, an effector of the RhoA pathway in the modulation of actin dynamics. In a report, it has been suggested that the overexpression of p57 determines the nuclear localization of LIMK1, with the subsequent loss of LIMK1-associated actin stress fibers [106]. Successively, Vlachos and colleagues demonstrated that the interaction of p57 with LIMK1 enhances the enzymatic activity of the kinase and thereby promotes the stabilization of the actin fibers through the phosphorylation of cofilin, the actin-severing protein [107]. In a further study, the knockdown of p57 delayed the migration of neurons in the cortical plate during mouse development; however, whether this resulted from the sequestration of LIMK1 was not investigated [108]. This bulk of evidence is consistent with the hypothesis of there being common mechanistic features of the CIP/Kip proteins, which exert antiproliferative functions in the nucleus but might participate in even pro-carcinogenic processes in the cytoplasm through the modulation of cytoskeletal dynamics.

The PCNA binding motif, homologous to that of p21, is located in the C-terminal moiety. Even though the affinity of the p57–PCNA binding is lower compared to that of the binding with p21, the functional importance of the complex is evident in the IMAGe (OMIM 614732) and Russel Silver (OMIM 180860) syndromes, characterized by undergrowth and developmental defects, where mutations in this domain have been identified [109].

The C-terminal domain of p57 has also been correlated with the apoptotic process. In normal cell lines, the protein seems to exert an anti-apoptotic function mainly related to the ability of the C-terminal domain of p57 to bind and inhibit JNK activities in a CDK-independent manner [20]. Particularly, in myoblasts, the binding of the protein to JNK competes and interferes with the interaction between JNK and c-Jun for the promotion of JNK/SAPK apoptotic signaling [20]. It is well known that the JNK/SAPK transduction cascade is preferentially activated by a large number of “stresses”, including UV radiation, ionizing radiation, and ROS (Reactive Oxygen Species) [110]. Through this pathway, the signals proceed towards the JNK-dependent activation of transcription factors that drive apoptotic processes and/or block cell proliferation. In this context, the inhibitory action of p57 could be an important mechanism by which the protein exerts its function on processes such as cell death and differentiation [20,111].

This evidence is in line with studies performed on *Cdkn1c^−/−^* mice that reported a high death rate at birth while, in those who survived, alterations of the differentiation programs, along with an increase in cell apoptosis, were observed [13,98]. Thus, the ability to regulate CDK activities with the N-terminal domain, together with the capacity to bind and to inhibit JNK/SAPK through the C-terminal domain, represents an important mechanism by which p57 controls cell cycle progression and cell death.

Comparably to the JNK pathway, p38 MAPK is also activated by a variety of cellular stressors, regulating processes from cell cycle checkpoints to cell differentiation and apoptosis [112]. Joaquin and colleagues, in 2012, reported the involvement of p57 in cell survival with various stimuli depending on p57 stabilization by p38 phosphorylation. The protein could then exert its CDK2 inhibitory activity, causing cell cycle arrest at the G1 phase and allowing the cells to respond to the stress condition. It has to be noted that the authors analyzed the phosphorylation of the mouse recombinant p57 protein transfected in HeLa cells, a human cervical cancer cell line, and confirmed the role of p57 in response to stressors in MEFs [103]. Unfortunately, human and mouse p57 proteins have structural differences that limit the extension of the results obtained in a mouse model to humans. On the contrary, the protein seems to exert a pro-apoptotic function in cancer. As a matter of fact, p57 was reported to enhance staurosporine-induced apoptosis in HeLa cells, and this effect was independent of the CDK-inhibitory activity of the protein [113]. The observation was confirmed in 2007 by a study demonstrating that p57 sensitized the cells to pro-apoptotic agents such as cisplatin, etoposide, and staurosporine. Intriguingly, the process involves a rapid translocation of p57 into mitochondria followed by the activation of the intrinsic pathway of apoptosis [107]. Additional experiments confirm a role for p57 in apoptosis, although the findings are frequently conflicting and dependent on the cell type and the experimental approach employed. In lung and colon carcinoma cell lines, p57 protects against doxorubicin-dependent cell death [114], while in other models, p73 induces apoptosis via a p57 increase. Specifically, p63 and p73 are members of the p53 family of transcription factors, and they are involved in cell cycle arrest and the induction of apoptosis. In a model of a teratocarcinoma cell line, the presence of p73 has been reported as being responsible for the induction of p57 and BAX expression, leading to apoptosis [115]. In the same way, the silencing of *CDKN1C* resulted in the suppression of the apoptosis mediated by p73 following cisplatin treatment in lung and colorectal cancer cell lines [116]. Interestingly, we recently reported the involvement of p57 in campthotecin-induced topoisomerase inhibition and the consequent DNA double-stranded break damage response [117]. However, depending on the cell model, the induction of p57 by anticancer drugs might be seen as a factor of resistance to the treatment.

Very few data are reported regarding a connection between p57 and autophagy. Recently, it has been shown that p57 accumulation decreased autophagy in hepatocarcinoma cells due to EGFR-targeted therapy. The mechanism by which p57 exerts this activity requires the activation of the PI3K/AKT/mTOR signaling pathway, which sensitizes cells to EGFR-inhibitor treatments [118].

p57 has been associated with cellular senescence. Particularly, the protein induces senescence when overexpressed in epithelial cells [119]. In hepatocellular carcinoma Hes1-depleted cells, p57 induces cellular senescence [120]. The inducible expression of p57 also drives senescence in astrocytoma cell lines [121]. Interestingly, senescence—which, traditionally, is associated with aging and pathological alterations—has recently been related to embryonic development. In placental tissues, where the expression of p57 is particularly high, the presence of the protein in placental extravillous trophoblasts after the invasion of the decidua has been considered a marker of senescence [122,123].

For the sake of clarity, Figure 2 also summarizes the biological processes modulated by p57 in the different cellular compartments.

#### 2.2.2. Regulation of p57 Cellular Levels

The expression of p57 changes during development, to become detectable only in a subset of human adult tissues. These pieces of evidence, together with the studies on knock-out mice and on the previously mentioned genetic syndromes, underline the role of this CDK regulator in embryogenesis, development, and aging.

The main mechanism of p57 abundance control occurs at the transcriptional level to ensure an accurate modulation of p57 dosage (reviewed in [124]). *CDKN1C* maps in a highly imprinted region with a very complex mechanism of gene transcription regulation [125,126]. Notably, the presence of multiple CpG islands upstream and downstream of the transcription start site favors the epigenetic control of *CDKN1C*, and the differentially methylated region on parental alleles leads the maternal allele to express the protein [127]. Thus, epigenetic control represents the main mechanism for the transcriptional regulation of *CDKN1C*. For example, during muscle development, the induction of p57 might require the action of MyoD. The latter can bind to a long-distance element located in the KvDMR1 imprinting control region, determining the release of a chromatin loop and favoring access to the promoter of *CDKN1C* [128].

The silencing of p57 expression has also been found in several tumors due to the genetic and epigenetic modulation of *CDKN1C* expression, although post-transcriptional regulation has been reported in some cell lines, as reviewed by Borriello and colleagues [19].

In addition, treatments with demethylating agents (such as 5′-azacytidine) and/or with histone deacetylase inhibitors (HDAC inhibitors, HDACIs) are able to enhance *CDKN1C* expression through multiple epigenetic and genetic mechanisms [129]. Specifically, treatment with HDACIs induces a chromatin remodeling that makes a minimal region, from −87 to −113 bp, of the *CDKN1C* promoter available for the binding of the transcription factor Sp1 (Several Stimulatory Protein-1), which positively regulates the expression of p57 in response to HDAC inhibitors [130,131,132]. However, *CDKN1C* expression can also be modulated through a change in the activity of several transcription factors. Indeed, the promoter region contains consensus sites for several transcription factors, including EGR1 (Early Growth Response 1), p63, p73, and GRE (glucocorticoid response element) [133,134]. EGR1 is a positive modulator of *CDKN1C* transcription [135,136]. Its action is reduced when it is associated with the chimeric protein PAX3-FoxO1, originated by the common chromosomal translocation characterizing some cases (alveolar subtype) of childhood rhabdomyosarcoma. The constitutively expressed oncogene PAX3-FoxO1, by inhibiting EGR1 activity, negatively regulates *CDKN1C* expression and induces an increase in the proliferation of myoblasts [135]. In some studies, the expression of the gene *CDKN1C* was also associated with the activity of p63 and p73, two proteins that, analogously to their family founder p53, are involved in the response to DNA damage. The observation that p63 knock-out mice exhibit a similar phenotype to *Cdkn1c^-/-^* mice suggests that p57 might be a p63 target [137]. Similarly, p73 controls p57 levels by the recognition of and binding to a consensus sequence in the promoter of *CDKN1C* [138]. A GRE has been identified in the human and mouse p57 promoters [139]. Glucocorticoids exert antiproliferative effects on numerous cell types, including HeLa cells. Several experimental studies suggest that dexamethasone treatment directly induces the transcription of *CDKN1C*, and the p57 protein can be considered to be involved in the glucocorticoid-induced antiproliferative effect [140]. In addition, it has been reported that other transcription factors can regulate p57 expression and might control CDKI levels: they are ETS (erythroblastosis virus E26 oncogene), a TATA box binding protein (TBP), OCT1 (octamer-binding transcription factor 1), NF1 (neurofibromin 1), HES1 (hairy and enhancer of split 1), and Herp2 (Hes-related Repressor Protein 2), which are NOTCH effectors [134].

Finally, besides epigenetic changes and signal-transduction modulation, regulation by microRNAs might be an additional mechanism contributing to p57 level control in a wide variety of solid and liquid tumors [141]. Particularly, miR25, miR221, and miR222 were reported as able to directly target CDKN1C RNA in gastric cancer [142], hepatocellular carcinoma (HCC), and human T-cell lymphoblastic lymphomas [143], and miR-92b has been reported as responsible for p57 downregulation in HCC tissues and cell lines, enhancing tumor radio-resistance to ionization radiation (IR)-based radiotherapy [144].

## 3. Are KIP Proteins Molecular Effectors of Polyphenols?

Polyphenols have, for many years, represented a class of natural molecules on which great interest has been concentrated in both basic and translational research. Although initially studied for their antioxidant activities, the discovery of the numerous phenotypic effects led to considering them as compounds with pleiotropic functions, including, particularly, anti-inflammatory, antiproliferative, anti-obesity, and anti-aging effects.

Mechanistically, polyphenols interact with many of the fundamental cellular processes by modulating the majority of the signal transduction pathways and significantly modifying the gene expression and the translation processes of the transcripts. One of the activities that has been reported in early studies on polyphenols is their ability to interfere with the cell division cycle. Indeed, the seminal study published in Science in 1997 by Pezzuto’s group [145], which exemplified a strong and clear effect of resveratrol on carcinogenesis and cell proliferation, remains exemplary. Subsequently, a plethora of studies have confirmed and extended the activities of polyphenols to the cell division cycle, activation of apoptosis, or induction of senescence.

A limitation of these studies exploring the biological activities of polyphenols regards the observation that most of them are based on cell lines as experimental systems, and the concentrations applied are in the tens or hundreds micromolar range. If, on the one hand, these features raise easy and routine criticisms as to the biological significance and clinical applicability of bioactive polyphenols, these, on the other hand, leave enormous space for further investigations in animal models and for clinical trials. We clearly expressed our point of view elsewhere of the strengths and weaknesses of using cell lines to study the bioactivities of polyphenols and their mechanism(s) of action and proposed guidelines in this direction [146].

As reported in the previous paragraphs, our knowledge of proteins involved in the control of cell cycle progression has significantly changed over the years, in relation to the discovery of new roles related to or completely independent of cell cycle control. Therefore, it seems interesting to critically review how polyphenols can interfere with regulation of p27 and p57, which, as previously described, represent two key proteins in the complex cycle control systems and in overall cell biology and physiology.

Although the first publication on this topic is dated to about 20 years ago, it is of interest to observe the evolution of the field and the attempts of many authors to find functional relationships and specificity between given phenolic compounds and their effects on cell phenotypes mediated by p27/p57. The following paragraphs are dedicated to those phenolic compounds that largely intercepted the scientists’ interest.

### 3.1. p27 and Polyphenols’ Phenotypic Effects

#### 3.1.1. Epigallocatechin-3-Gallate (EGCG)

**Figure 3:**
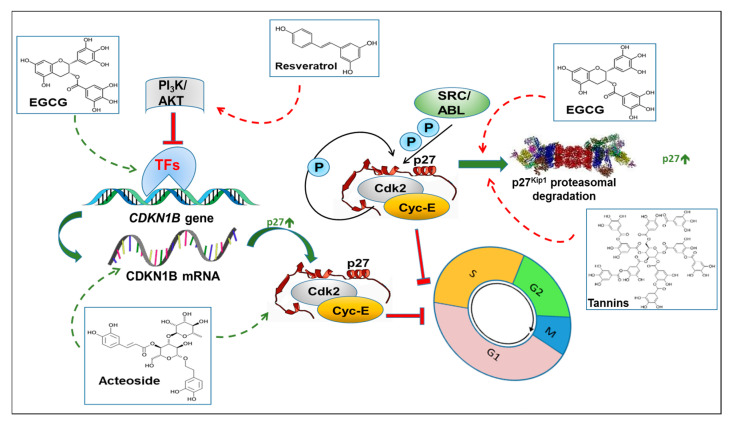
Schematic representation of selected polyphenols that interfere with p27 expression and activity (see Table 1 and text for details). Epigallocatechin-3-gallate (EGCG) increases the expression of p27, acting at multiple levels: stimulating the activity of its transcription factors (TFs, mainly FoxO) or inhibiting its proteasomal degradation. Resveratrol inhibits the pathway (PI_3_K/AKT) that negatively regulates the transcription of the *CDK1B* gene. Tannins also act by directly binding and inhibiting the proteasome-dependent degradation of p27. Acteoside increases CDK1B mRNA synthesis and favors the binding of p27 to CDK4/6, contributing to the reduction of their kinase activity. The increased expression of p27 potentiates its inhibitory effect on cell cycle progression, blocking malignant cells at the G1/S transition. Dashed red and green arrows indicate inhibition and activation, respectively, mediated by “indirect” interactions between the indicated polyphenols and p27-regulating factors.

Early studies indicated that EGCG (Figure 3; Table 1) induced G0/G1-phase cell cycle arrest and apoptosis in A431, a human epidermoid carcinoma cell line, in a concentration range of 10–80 μg/mL (about 20–170 μM). This effect coincided with a significant dose- and time-dependent upregulation of p27 and of other cell division cycle inhibitors, such as p21, p16INK4A, and p18INK4C; decreased expression of cyclin D1; and inhibition of CDK2, CDK4, and CDK6 kinase activities [147]. A similar effect was evidenced in both androgen-sensitive (LNCaP) and androgen-insensitive (DU145) human prostate carcinoma cells at comparable concentrations (about 10–80 μM). In this case, the block at G1–S transition and the induction of G1 arrest were followed by apoptotic induction [148]. Later, in cervical tumor cells (HeLa, Caski, and SiHa), it was reported that the cell cycle arrest and apoptotic induction triggered by EGCG (10–80 μM) was mediated by a specific EGCG-dependent suppression of EGFR activation, resulting in the inhibition of ERK1/2 and AKT enzymatic activities. These events were associated with downstream changes, such as increased p53, p21, and p27 levels and reduced CDK2 kinase activity and cyclin E levels [149]. Evidence has been published for the capability of EGCG to act in combination therapy. BT474 and JIMT-1 human breast cancer cells are both resistant to trastuzumab, the humanized monoclonal antibody against HER2, the human epidermal growth factor receptor 2, which is overexpressed in about 30% of breast cancers and considered a marker of poor prognosis. In these cell lines, treatment with EGCG (45–90 μM) inhibited cell growth and induced apoptosis through a pathway that included the reduced phosphorylation of AKT-Ser473, which freed FoxO3a to allow it to translocate into the nucleus, where it could activate target genes, including *CDKN1B*, responsible for cell cycle arrest. FoxO proteins are evolutionarily conserved transcription factors whose inactivation is frequently observed in human cancers [150]. Accordingly, in trastuzumab-resistant breast cancer cell lines, the EGCG-dependent expression of p27 closely correlated with FoXO3a nuclear accumulation [151].

The involvement of p27 in mediating the anticancer effects of EGCG has been corroborated by other reports based on the administration of green tea extract, which is rich in polyphenols, including EGCG. As an example, in a rat model of mammary tumorigenesis induced by 7,12-dimethylbenz(a)anthracene (DMBA), the administration of a certified green tea extract (containing 11.79% EGCG) given to rats as a unique fluid source (0.3%) for 17 weeks significantly reduced the DMBA-induced tumor burden and invasiveness and increased the latency of the first tumor. The effect of the green tea beverage was confirmed in Hs578T and MDA-MB-231 triple-negative breast cancer cell lines, where 80–160 μg/mL of the extract or 90–180 μM EGCG similarly inhibited cell proliferation and induced apoptosis. At least in cancer cell lines, the antiproliferative effect of EGCG was associated with the upregulation of p27. Particularly, lower doses of EGCG slowed cell growth by increasing p27 levels, which correlated with the arrest of cells at the G1/S phase transition. Higher doses enhanced apoptosis [111]. Others suggested a different mode of action for some tea polyphenols, including EGCG [152]. In this case, they noted that tea poMSTOlyphenols containing ester bonds (e.g., EGCG, EGC (2)2epigallocatechin-3-gallate, GCG, (2)2gallocatechin-3-gallate CG, and (2)2catechin-3-gallate) could inhibit proteasome activity, based on evidence that the same ester bonds were present in conventional proteasome inhibitors (e.g., lactacystin β-lactone). In fact, these polyphenols inhibited, both in vitro and in vivo, the chymotrypsin-like activity of the proteasome (the IC50 values for ECG, GCG, and CG were 194, 187, and 124 nM, respectively). Because of this effect, EGCG, in several malignant cell lines, induced the “indirect” accumulation of two canonical proteasome substrates, p27 and IkappaB-alpha, which, in turn, mediated growth arrest in the G1 phase of the cell division cycle [152].

#### 3.1.2. Resveratrol

In 2002, for the first time, Reddy’s group evidenced the double nature of resveratrol (RSV) in regulating cell cycle progression and its cell-type specificity [153] (Figure 3; Table 1). In fact, only in androgen-sensitive LNCaP cells, but not in androgen-independent DU145 prostate cancer cells or in NIH3T3 fibroblasts, did RSV increase DNA synthesis, pushing cells into the S phase; however, this effect was measurable only at lower RSV concentration (5–10 μM) and was mediated by a decrease in the nuclear levels of p21 and p27. On the contrary, at concentrations higher than 20 μM, RSV inhibited DNA synthesis, creating a “collision course” between two important cell cycle phases, e.g., the entry of cells into the S phase and progression through the S phase. The authors speculated that this behavior of RSV could be exploited in the chemotherapy of prostate cancer by accelerating the proliferation of cancer cells and making them more sensitive to chemotherapeutic agents and ionizing radiation [154]. Later, by comparing the apoptotic and cell cycle effects of RSV on LNCaP versus other prostate-derived cells, namely, PZ-HPV-7 (non-tumorigenic line) and PC-3 (androgen-insensitive cancer cell line), Benitez et al. [155] reported that RSV, at 1–150 μM concentrations, differently affected cell cycle progression in these cell lines. In fact, the molecule blocked LNCaP cells almost exclusively in G1/S, while a significant percentage of PC-3 cells were also arrested in G2/M, with p21 and p27 expression increased by RSV only in LNCaP cells. Since LNCaP and PC-3 cells are representative of two different types of prostate cancer, it can be speculated that RSV triggered different regulators depending on the cell lines’ androgen sensitivity and androgen insensitivity. In support of this view, it has been reported that methyl ether analogs of RSV, present in edible plants, exhibited differential effects on LNCaP. As an example, RSV, pinostilbene, and pterostilbene induced cell cycle arrest at G1/S, while resveratrol trimethylether led to G2/M block [156]. A new wave of novelty regarding the effects of RSV on cell cycle regulation emerged in the biennium 2010–11, when several papers were published on the regulatory effects of RSV on the PI_3_K/AKT/FoxO pathway. As reported above, FoxO proteins are transcription factors whose inactivation is frequently observed in cancers [150]. In LNCaP cells, RSV (10–20 μM) inhibited the PI_3_K/AKT pathway and stabilized the levels and enhanced the transcriptional activity of FoxO, finally resulting in the upregulation of its target genes (TRAIL, TRAIL-R1/DR4, TRAIL-R2/DR5, Bim, p27, and cyclin D1). Consequently, the overexpression of FoxO genes (*FKHR, FKHRL1*, and *AFX*) enhanced these effects, while their inactivation abolished the RSV-induced expression of TRAIL, TRAIL-R1/DR4, TRAIL-R2/DR5, Bim, and p27. RSV had no direct effect on the expression of FoxO [157]. This finding was confirmed in mice xenografted with PC-3 cells, where RSV (30 mg/kg) administered through gavage alone or in combination with TRAIL inhibited tumor growth and angiogenesis; upregulated the expression of TRAIL-R1/DR4, TRAIL-R2/DR5, BAX, and p27; and inhibited the expression of Bcl-2 and cyclin D1 [158]. Comparable data were obtained for different cancer types, e.g., pancreatic cancer and leukemia. In the former, RSV reduced cell proliferation and induced caspase-3-dependent apoptosis in several pancreatic cancer cell lines (PANC-1, MIA PaCa-2, Hs766T, and AsPC-1). Cell cycle arrest was mediated by the upregulation of p21 and p27 expression and inhibition of cyclin D1 expression. When PANC-1 cells were orthotopically implanted in Balb C nude mice, RSV treatment (0–60 mg/kg body weight, through gavage) induced the upregulation of Bim, p27, p21, and cleaved caspase-3 and inhibited the expression of PCNA. In addition, RSV reduced the phosphorylation of PI_3_K-Tyr458, AKT-Ser473, FoxO-Ser256, and FoxO3a-Ser253 without significant changes in their total protein levels [159]. In a panel of leukemic cells (K562, U937, NB4, Daudi, and Raji), non-toxic concentrations of RSV (5–20 μM) reduced the cytotoxicity of proteasome inhibitors. RSV, in combination with MG132 (a potent cell-permeable proteasome inhibitor that reduces the degradation of ubiquitin-conjugated proteins in mammalian cells), induced cell cycle arrest at the G1/S phase via p27 due to increased FoxO1-dependent expression at the transcriptional level. Knocking down p27 with siRNA almost abolished the protective effects of RSV. The authors concluded that MG132 and RSV synergistically induced p27 through the enhanced recruitment of FoxO1 to the *CDKN1B* promoter [160]. As mentioned above, p27 is strongly upregulated by proteasome inhibitor treatment, since the major mechanism of p27 level control is through ubiquitin/proteasome-dependent degradation acting in the nucleus and allowing the completion of the S/G2 phases [44].

In human colon cancer cells, RSV at high concentrations (100–150 μM) suppressed IGF-1-induced cell proliferation and increased apoptosis following G1/S-phase cell cycle arrest through p27 stimulation and cyclin D1 suppression [161]. In human malignant pleural mesothelioma cells (MSTO-211H), RSV (0–60 μM) decreased cell viability and increased apoptosis, with an IC50 of about 16 μM [162]. This study is of particular interest since a direct interaction between RSV and the Sp1 transcription factor was reported. The binding of Sp1 to G-C-rich promoters was inhibited by RSV, resulting in the reduced expression of cancer-related genes (p27, p21, cyclin D1, and Mcl-1) under the control of Sp1. The data were also confirmed in BALB/c athymic (nu+/nu+) mice injected with MSTO-211H cells and treated with RSV (20 mg/kg daily for 4 weeks). In this model, Sp1 expression was inhibited, with a parallel induction of apoptosis [162]. The unsolved paradox emerging from this work was the strong and unexpected reduction of p27, not clearly commented on and explained by the authors.

Several examples have been published on the antiproliferative effects of RSV when combined with other natural or synthetic agents. In Caco-2 cells, derived from a human colon adenocarcinoma, RSV (50 μM) associated with butyrate (2 mM) enhanced the induction of p21 and attenuated the expression of p27 [163]. These data suggest that RSV magnified the differentiating effects of butyrate on Caco-2 cells, and this capacity was mediated by p21 rather than p27. In several lung cancer cell lines—namely, A549, EBC-1, and Lu65—RSV inhibited cell growth with an ED50 in the range of 5–10 μM. Although RSV did not synergize with paclitaxel (Taxol^®^) in the same cell lines, pre-treatment with RSV at a relatively low concentration (10 μM) for three days significantly enhanced the subsequent apoptotic and antiproliferative effects of paclitaxel. Among the biochemical markers triggered by the combined effects of RSV and paclitaxel (p21, p27, E-cadherin, EGFR, and Bcl-2), only p21 expression was increased, approximately 4-fold [164]. More recently, RSV (35–47 μM) in combination with docetaxel (10–31 nM) modulated apoptosis and cell cycle progression in the C4-2B and DU-145 prostate cancer cells, differently sensitive to docetaxel. The combined treatment upregulated and downregulated the pro-apoptotic and anti-apoptotic genes, respectively, in both cell lines. In C4-2B cells, more sensitive to docetaxel, p21 and p27 levels were increased, leading to cell cycle arrest at the G1/S transition. An additional block was evidenced in the G2/M phase, matching with the suppression of CDK1 activity and cyclin B1 expression, suggesting that the cells that passed the G1/S checkpoint were blocked in G2/M. These effects were less pronounced in DU145 cells due to their reduced sensitivity to docetaxel [165].

Unexpectedly, in non-malignant cells, e.g., vascular smooth muscle cells, RSV arrested cells in the S phase, decreasing the expression of p21 and p27 and increasing the phosphorylation of the Rb protein [166]. Other authors confirmed that, in the same cellular model, RSV blocked the cell cycle in the G1 phase, downregulated the levels of cyclin D1 and CDKs, and upregulated the expression of p21, but did not increase p27 [167]. This observation leaves space for future investigation of the role of RSV in the so-called “collision course” between two important cell cycle phases, e.g., the entry of cells into the S phase and progression through the S phase.

#### 3.1.3. Other Polyphenols

Hydrolysable tannins, generally called tannic acid, represent a class of plant-derived polyphenols with molecular weights ranging between 500 and 3000 daltons containing 6 to 9 ester bonds. Tannic acid, similarly to a few other ester bond-containing tea polyphenols (e.g., EGCG), was able to inhibit the chymotrypsin-like activity of both purified 20S proteasome and Jurkat cell 26S proteasome. Since, as previously described, p27 is among the targets of the ubiquitin/proteasome-mediated degradation pathway, it is plausible that the direct inhibition of the 26S proteasome by tannic acid generates the accumulation of p27 and the consequent induction of G1/S arrest and cell death [168] (Figure 3; Table 1).

Guggulsterone (4,17(20)-pregnadiene-3,16-dione) is a phenolic compound derived from the gum resin of the *Commiphora mukul* tree and used in traditional medicine as a remedy against inflammatory diseases. This molecule inhibited the cell growth of a wide variety of tumor cells (but not normal human fibroblasts), blocking them in G1/S, with the downregulation of cyclin D1 and upregulation of p21 and p27. It is of interest that the effects of guggulsterone increased sensitivity to chemotherapy in drug-resistant cancer cells, favoring apoptotic cell death [169].

Acteoside, the alpha-L-rhamnosyl-(1->3)-β-d-glucoside of hydroxytyrosol, at approximately 30 μM concentration, arrested HL-60 human promyelocytic leukemia cells in the G1 phase, inducing differentiation via a mechanism that not only increased the mRNA and protein levels of p27 and p21 but also favored their binding to CDK4 and CDK6, contributing to the reduction of their kinase activities [170] (Figure 3; Table 1).

Carnosic acid is a polyphenolic compound present in rosemary, oregano, and other culinary herbs. In human myeloid leukemia cells (HL-60 and U937), at 2.5–10 μM (EC50, about 6–7 μM), cell proliferation was inhibited without the induction of apoptosis (Figure 3; Table 1). The G1/S arrest coincided with an increase in the p21 and p27 proteins [171]. Carnosic acid is easily converted to carnosol by oxidation. Carnosol inhibited cell viability (25–100 μM) in the MDA-MB-231 cell line through multiple mechanisms. The block in the G2/M phase of the cell cycle was mediated by the increased expression of p21 and downregulation of p27. Beclin1-independent autophagy and apoptosis were also induced, with autophagy that preceded apoptosis. At a lower and non-cytotoxic concentration (25 μM), carnosol stimulated a limited production of ROS, enough to activate γH2AX (Ser139-phosphorylated histone H2AX), a variant of histone H2A that plays an important role in the cellular response to DNA double-stranded breaks, and to induce autophagy. At a higher concentration (100 μM), carnosol induced a massive production of ROS, responsible for triggering autophagy followed by the activation of both intrinsic and extrinsic apoptotic pathways [172]. The role of cell cycle arrest in this scenario remains to be clarified.

Theaflavin-3,3′-digallate is a unique polyphenol present in black tea produced by the fermentation of EGCG and epicatechin gallate. In OVCAR-3 human ovarian carcinoma cells, this compound inhibited cell growth, acting on parallel mechanisms: (1) at 20–30 μM concentrations, it enhanced the phosphorylation of the checkpoint kinase 2 (Chk2), which, in turn, activated intrinsic apoptosis independently of p53, increasing the Bax/Bcl-2 ratio; (2) theaflavin-3,3′-digallate (20 μM) caused G0/G1 arrest with an expected increase in p27 levels and a dramatic downregulation of CDK4, Cyclin D1, p-Rb, and Rb [173]. It remains to be explained if and how these two events are independent or functionally related.

Hydroxytyrosol (2-(3,4-dihydroxyphenyl)ethanol), one of the most studied polyphenols from olive oil, is known for its anti-inflammatory, antioxidant, and anticancer activities. Among these, hydroxytyrosol was able to downregulate EGFR (epidermal growth factor receptor) expression and inhibit cell growth in both colon carcinoma cell lines and xenograft models with a mechanism that mimics the effect of the EGFR inhibitor cetuximab. In recent work, Terzuoli et al. [174] demonstrated that the association between hydroxytyrosol (10 μM) and cetuximab (1 μg/mL) was 10 times more efficient in reducing cell growth in HT-29 and WiDr colon cancer cells than the single compounds. The efficacy of the combined effect was mediated by cell cycle arrest at the G2/M phase, associated with the induction of both p21 and p27. This event, with a mechanism still not fully clarified, induced caspase-independent apoptosis and autophagy activation, evidenced by a strong increase in Beclin-1 levels [174]. Previous studies carried out in the promyelocytic cell line HL60 demonstrate that the cell growth inhibition and the apoptosis induction activities require the presence of two ortho-hydroxyl groups on the phenyl ring [175].

Finally, a mention is deserved by a recent study focused on the anticancer capacity of polyphenol metabolites generated through the colonic microflora-dependent degradation of their parental aglycones, which are generally more unstable and present at nanomolar concentrations in the blood stream. 2,4,6-trihydroxybenzoic acid (2,4,6-THBA) can be generated by the degradation and subsequent oxidation of most flavonoids (anthocyanins, flavonols, flavones, and flavanols). In cells expressing a functional SLC5A8 (a monocarboxylic acid transporter), this metabolite was able to accumulate in the cells at pharmacological concentrations (250–1000 μM), slowing down the rate of cell proliferation and massively increasing both p21 and p27. In addition, molecular docking studies indicated that 2,4,6-THBA was able to directly bind to CDKs 1, 2, and 4, and this prediction was confirmed by the inhibition of their kinase activities in in vitro CDK assays employing the purified enzymes [176].

### 3.2. p57: A Promising (Putative) Actor in Polyphenols’ Mechanism of Action

**Figure 4:**
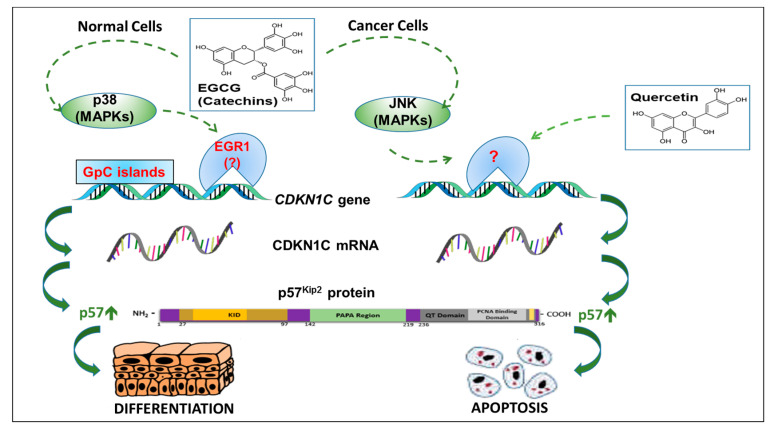
Schematic representation of how selected polyphenols can interact with p57 expression and activity (see Table 1 and text for details). EGCG, as well as other catechins, upregulates the expression of p57 in normal cells, inducing differentiation (e.g., human keratinocytes), acting on *CDKN1C* gene transcription via members of the MAPK family, possibly p38. In cancer cells, the increased expression of p57 leads to apoptosis through a pathway probably involving JNK. Quercetin also induces apoptosis, upregulating *CDKN1C* gene expression via uncharacterized factors. Dashed green arrows indicate activation mediated by “indirect” interactions between the indicated polyphenols and p57-regulating factors.

The literature regarding the regulation of p57 by polyphenols is very limited and largely derives from the work performed by Hsu and Schuster’s group. These authors firstly demonstrated that polyphenols from green tea, either in the form of a mixture of the four major catechins (epicatechin, epigallocatechin, epicatechin-3-gallate, and EGCG) or as purified EGCG, were able to induce the transient expression of p57 in normal human epidermal keratinocytes [177] (Figure 4; Table 1). The p57 induction resulted in the stimulation of multiple survival pathways including cell differentiation [177,178]. They subsequently reported that green tea catechins promoted the “re-energization” of aged keratinocytes, while the induction of p57 stimulated the differentiation of the keratinocytes in the basal layer of the epidermis. These effects were interpreted as combined, positive effects of green tea catechins in accelerating wound healing and the regeneration of new skin tissues via p57 [179]. On the contrary, in cancer cells, the same green tea constituents generated a significantly different response. In the oral carcinoma cell lines SCC25 and OSC2, EGCG induced apoptosis and inhibited cell growth and invasion without altering the levels of p57 expression [177,180] (Figure 4; Table 1). In addition, the retroviral transfection of p57 in oral carcinoma cells increased resistance to catechin-induced apoptosis, suggesting that p57 was a key pro-survival factor counteracting the apoptosis induced by green tea polyphenols [178]. In the following years, more details were added to the hypothesis that catechins activate differentiation in normal epidermal keratinocytes and apoptosis in tumor cells. Using NHEK (normal human epidermal keratinocytes), it was reported that EGCG induced differentiation by stimulating the expression of both p57 and caspase 14, a factor not directly involved in the typical apoptotic process but associated with the terminal differentiation of NHEK and barrier formation. The overexpression of p57 significantly preceded and was required for the increase in the expression level of caspase 14, suggesting that, in NHEK cells, p57 acted as a regulator of caspase 14 expression [181]. The precise mechanisms responsible for the upregulation of p57 by EGCG are not completely understood. With MAPK proteins (p38, ERK, and JNK) being associated with EGCG signaling [182], the authors also demonstrated that the EGCG-mediated induction of p57 in keratinocytes required p38 MAPK activity—since the use of SB203580, a p38 inhibitor, prevented p57 accumulation—but was independent of JNK or MEK activity [21].

On the contrary, in oral carcinoma cell lines (OSC2), EGCG induced a rapid activation of JNK followed by caspase-dependent apoptosis. The chemical inhibition of JNK activity abolished the pro-apoptotic effect of EGCG, and an OSC2 subclone expressing a high level of p57 failed to develop tumors in xenograft mice [21]. These results suggest that the loss or reduced expression of endogenous p57 is associated with malignant transformation in oral carcinomas. The effect can be bypassed by JNK activation in response to EGCG treatment or overexpressing p57. The latter event promotes differentiation and abolishes the EGCG-dependent activation of JNK and caspase 3. In other words, green tea catechins can act as a switch for different MAPK proteins, inducing differentiation or apoptosis depending on the presence and level of expression of p57. In normal cells, catechins induce cell differentiation via p38, leading to the overexpression of p57, which, in turn, blocks the JNK-dependent apoptotic pathway stimulated by catechins. In cancer cells, the low/null expression of p57 frees catechins from the possibility of activating JNK and inducing apoptosis.

In conclusion, although still speculative, the transient expression of p57 in normal human epidermal keratinocytes mediated by EGCG/catechins may suggest a role of these polyphenols in the “re-energization” of aged keratinocytes. This observation can open the door to investigate in more detail the possibility that p57 participates in mediating the anti-aging effects of polyphenols. The field appears to be extremely promising since, as commented elsewhere [183], the molecular mechanisms of the beneficial effects of polyphenols in aging and non-communicable diseases can be approached by administering nutritional (not pharmacological) doses, such as those present in polyphenol-enriched diets and/or nutraceuticals/functional foods.

Only few data are available on the ability of other polyphenols to promote *CDKN1C* transcription in cancer cell lines, favoring cell cycle arrest and apoptosis. MCF7 breast cancer cells treated with quercetin showed an increase in p57 in parallel with p53 and the activation of the apoptotic pathway [184] (Figure 4; Table 1). In previous work, we reported that resveratrol exerts strong antiproliferative activity in the micromolar range, upregulating the EGR1 level [175]. Later, in a second report, we evaluated the effect of resveratrol on the expression of *CDKN1C* in K562 cells. Although it was confirmed that resveratrol induced an increase in EGR1, the compound did not influence the protein amount, suggesting that, at least in the used cell model, resveratrol-mediated EGR1 induction is not involved in the modulation of *CDKN1C* transcription [131] 

## 4. Conclusions and Future Directions

We learned from this review that both of the “main actors”, i.e., Kip proteins and polyphenols, share two key qualities: strong promiscuity and pleiotropy. In fact, although p27/p57 originally became popular in the field of cell cycle regulation for their pivotal role as CDKIs, now, their CDK-unrelated functions are increasingly maturing towards the control of multiple complex processes that include apoptosis, autophagy, DNA-damage repair, gene transcriptional control, cytoskeleton remodeling, and many others, as we largely described in the above sections. Analogously, polyphenols are, “by definition”, pleiotropic compounds, being able to hit and modulate multiple independent or complementary pathways that control the same key processes regulating cellular homeostasis and implicated in the physiopathology of complex diseases, such as cancers and cardiovascular and neurodegenerative disorders. In the present work, we attempted to harvest evidence for the existence of possible interplay between polyphenols and p27/p57 functions. We can conclude that, although this topic remains in its infancy and requires further studies, some interesting and promising outcomes are already emerging.

As it is a key issue, we need to understand if the described changes in the cellular levels/activity of p27/p57, observed in many cellular models, are the “cause” or the “consequence” of the phenotypical effects of the polyphenol treatments. The latter appears to be the easiest answer. It is well known that polyphenols can arrest the cell cycle in cancer cells [185], and p27/p57 are part of the downstream mechanisms controlling this process. Therefore, it is conceivable to expect an increase in their levels, together with those of other possible inhibitors of cell proliferation, because of the upstream events triggered by polyphenols, ending up with cell cycle arrest. This simplistic explanation attributes a marginal and non-specific role to the interplay between polyphenols and p27/p57. This possibility cannot be excluded in the absence of strong evidence indicating i. direct binding between specific phenolic compounds and p27 or p57, or ii. biochemical and/or genetic demonstrations that the effects of polyphenols on cell cycle arrest are abolished/reduced by inhibiting the expression of p27/p57. However, the circumstantial evidence presented in this review is being built upon regarding the existence of more specific mechanisms activated by polyphenols regulating p27/p57 functions. Among this are the following: i. the observation that in some experimental settings, p27 expression is downregulated by polyphenols differently from what it is expected in terms of cell cycle arrest (summarized in Table 1); ii. the consolidated demonstration that EGCG and other polyphenols can specifically stimulate p27 accumulation by inhibiting the ubiquitin/proteasome-mediated degradation pathway, with the consequent induction of G1/S arrest and cell death; iii. the data obtained in our laboratories suggesting that a natural extract enriched in polyphenols arrests cell growth and activates autophagy via changes in p27 phosphorylation and its cellular localization with mechanisms probably independent from the cell-cycle-regulatory role of p27 (data not reported). This observation, although preliminary, points towards the novel role of p27 in autophagy commented on above, which will certainly represent a target of new studies in the field of Kip proteins.

In conclusion, shedding light on the biological mechanisms regulating the interplay between Kip proteins and polyphenols is much more complex than it appears, and future studies will be open to new and unexpected perspectives.

## Figures and Tables

**Table 1:** Studies reporting the effects of selected polyphenols on Kip proteins.

Compound	Experimental Model	Concentration	Effect on p27/p57	Reference
EGCG	A431 (epidermoid carcinoma)	20–170 μM	p27🡹	[147]
	LNCaP (prostate carcinoma) DU145 (prostate carcinoma) HeLa (cervical adenocarcinoma) Caski (epidermoid cervical carcinoma) SiHa (squamous cell carcinoma)	10–80 μM	p27🡹 apoptosis🡹	[148,149]
Green tea extract	Hs578T (mammary carcinoma) MDA-MB-231 (mammary adenocarcinoma)	80–160 μg/mL (90–180 μM EGCG)	p27🡹 apoptosis 🡹	[111]
Resveratrol	LNCaP (prostate carcinoma)	5–10 μM	nuclear p27🡻	[154]
	LNCaP	> 20μM	p27🡹	[155]
	Xenografts (PC-3 cells)	30 mg/kg	p27🡹	[158]
	Leukemic cells	5–20 μM	p27🡹	[160]
	MSTO-211H (pulmonary mesothelioma) Xenografts (MSTO-211H)	60 μM 20 mg/kg	p27🡻 apoptosis 🡹	[162]
	Vascular smooth muscle cells	1–100 μM	p27🡻 cell cycle arrest G1/S	[166]
Guggulsterone	Leukemia, Myeloma, Head and Neck Carcinoma, Lung, Melanoma, Breast, Ovarian, Embryonic Kidney cell lines	1–50 μM	p27🡹 cell cycle arrest G1/S	[169]
Acteoside	HL-60 (acute promyelocytic leukemia)	30 μM	p27🡹 cell cycle arrest G1/S	[170]
Carnosic acid	HL-60 U937 (histiocytic lymphoma)	2.5–10 μM	p27🡹 cell cycle arrest G1/S	[171]
Carnosol	MDA-MB-231	25–100 μM	p27🡻 cell cycle arrest G2/M	[172].
Theaflavin-3,3’-digallate	OVCAR-3 (ovarian adenocarcinoma)	20–30 μM	p27🡹 apoptosis 🡹 cell cycle arrest G0/G1	[173]
Hydroxytyrosol + Cetuximab	HT-29 (colorectal adenocarcinoma) WiDr (colorectal adenocarcinoma)	10 μM 1 μg/mL	p27🡻 apoptosis 🡹 autophagy🡹 cell cycle arrest G2/M	[174]
Green tea extract EGCG	NHEK (human keratinocytes)	200 μg/mL 50–200 μM	p57🡹 differentiation🡹	[177,178,181]
EGCG	SCC25 (squamous cell carcinoma) OSC2 (squamous cell carcinoma)	15–200 μM	P57=🡹 apoptosis 🡹	[177,180]
Quercetin	MCF7 (breast adenocarcinoma)	10–175 μM	p57🡹 apoptosis🡹	[184]

🡹 and 🡻 symbols indicate increase and decrease, respectively of the indicated protein/process.

## References

[1] Ross J.A., Kasum C.M. (2002). Dietary flavonoids: Bioavailability, metabolic effects, and safety. Annu. Rev. Nutr..

[2] Dennis K.K., Go Y.M., Jones D.P. (2019). Redox Systems Biology of Nutrition and Oxidative Stress. J. Nutr..

[3] Meeran S.M., Katiyar S.K. (2008). Cell cycle control as a basis for cancer chemoprevention through dietary agents. Front. Biosci..

[4] Ramos S. (2008). Cancer chemoprevention and chemotherapy: Dietary polyphenols and signalling pathways. Mol. Nutr. Food Res..

[5] Sherr C.J., Roberts J.M. (1995). Inhibitors of mammalian G1 cyclin-dependent kinases. Genes Dev..

[6] Fotedar R., Fitzgerald P., Rousselle T., Cannella D., Doree M., Messier H., Fotedar A. (1996). p21 contains independent binding sites for cyclin and cdk2: Both sites are required to inhibit cdk2 kinase activity. Oncogene.

[7] Hashimoto Y., Kohri K., Kaneko Y., Morisaki H., Kato T., Ikeda K., Nakanishi M. (1998). Critical role for the 310 helix region of p57(Kip2) in cyclin-dependent kinase 2 inhibition and growth suppression. J. Biol. Chem..

[8] Russo A.A., Jeffrey P.D., Patten A.K., Massague J., Pavletich N.P. (1996). Crystal structure of the p27Kip1 cyclin-dependent-kinase inhibitor bound to the cyclin A-Cdk2 complex. Nature.

[9] Besson A., Dowdy S.F., Roberts J.M. (2008). CDK inhibitors: Cell cycle regulators and beyond. Dev. Cell.

[10] Macleod K.F., Sherry N., Hannon G., Beach D., Tokino T., Kinzler K., Vogelstein B., Jacks T. (1995). p53-dependent and independent expression of p21 during cell growth, differentiation, and DNA damage. Genes Dev..

[11] Kreis N.N., Louwen F., Yuan J. (2019). The Multifaceted p21 (Cip1/Waf1/CDKN1A) in Cell Differentiation, Migration and Cancer Therapy. Cancers.

[12] Borriello A., Cucciolla V., Oliva A., Zappia V., Della Ragione F. (2007). p27Kip1 metabolism: A fascinating labyrinth. Cell Cycle.

[13] Yan Y., Frisen J., Lee M.H., Massague J., Barbacid M. (1997). Ablation of the CDK inhibitor p57Kip2 results in increased apoptosis and delayed differentiation during mouse development. Genes Dev..

[14] Lee M.H., Reynisdottir I., Massague J. (1995). Cloning of p57KIP2, a cyclin-dependent kinase inhibitor with unique domain structure and tissue distribution. Genes Dev..

[15] Casini T., Pelicci P.G. (1999). A function of p21 during promyelocytic leukemia cell differentiation independent of CDK inhibition and cell cycle arrest. Oncogene.

[16] Duquesnes N., Callot C., Jeannot P., Daburon V., Nakayama K.I., Manenti S., Davy A., Besson A. (2016). p57 (Kip2) knock-in mouse reveals CDK-independent contribution in the development of Beckwith-Wiedemann syndrome. J Pathol..

[17] Sharma S.S., Pledger W.J. (2016). The non-canonical functions of p27 (Kip1) in normal and tumor biology. Cell Cycle.

[18] Minervini G., Lopreiato R., Bortolotto R., Falconieri A., Sartori G., Tosatto S.C.E. (2017). Novel interactions of the von Hippel-Lindau (pVHL) tumor suppressor with the CDKN1 family of cell cycle inhibitors. Sci. Rep..

[19] Borriello A., Caldarelli I., Bencivenga D., Criscuolo M., Cucciolla V., Tramontano A., Oliva A., Perrotta S., Della Ragione F. (2011). p57 (Kip2) and cancer: Time for a critical appraisal. Mol. Cancer Res..

[20] Chang T.S., Kim M.J., Ryoo K., Park J., Eom S.J., Shim J., Nakayama K.I., Nakayama K., Tomita M., Takahashi K. (2003). p57KIP2 modulates stress-activated signaling by inhibiting c-Jun NH2-terminal kinase/stress-activated protein Kinase. J. Biol. Chem..

[21] Yamamoto T., Digumarthi H., Aranbayeva Z., Wataha J., Lewis J., Messer R., Qin H., Dickinson D., Osaki T., Schuster G.S. (2007). EGCG-targeted p57/KIP2 reduces tumorigenicity of oral carcinoma cells: Role of c-Jun N-terminal kinase. Toxicol. Appl. Pharmacol..

[22] Matsuoka S., Edwards M.C., Bai C., Parker S., Zhang P., Baldini A., Harper J.W., Elledge S.J. (1995). p57KIP2, a structurally distinct member of the p21CIP1 Cdk inhibitor family, is a candidate tumor suppressor gene. Genes Dev..

[23] Adkins J.N., Lumb K.J. (2002). Intrinsic structural disorder and sequence features of the cell cycle inhibitor p57Kip2. Proteins.

[24] Bienkiewicz E.A., Adkins J.N., Lumb K.J. (2002). Functional consequences of preorganized helical structure in the intrinsically disordered cell-cycle inhibitor p27(Kip1). Biochemistry.

[25] Martinelli A.H.S., Lopes F.C., John E.B.O., Carlini C.R., Ligabue-Braun R. (2019). Modulation of Disordered Proteins with a Focus on Neurodegenerative Diseases and Other Pathologies. Int. J. Mol. Sci..

[26] Schmetsdorf S., Gartner U., Arendt T. (2007). Constitutive expression of functionally active cyclin-dependent kinases and their binding partners suggests noncanonical functions of cell cycle regulators in differentiated neurons. Cereb Cortex.

[27] Polyak K., Kato J.Y., Solomon M.J., Sherr C.J., Massague J., Roberts J.M., Koff A. (1994). p27Kip1, a cyclin-Cdk inhibitor, links transforming growth factor-beta and contact inhibition to cell cycle arrest. Genes Dev..

[28] Slingerland J.M., Hengst L., Pan C.H., Alexander D., Stampfer M.R., Reed S.I. (1994). A novel inhibitor of cyclin-Cdk activity detected in transforming growth factor beta-arrested epithelial cells. Mol. Cell. Biol..

[29] Toyoshima H., Hunter T. (1994). p27, a novel inhibitor of G1 cyclin-Cdk protein kinase activity, is related to p21. Cell.

[30] Ou L., Ferreira A.M., Otieno S., Xiao L., Bashford D., Kriwacki R.W. (2011). Incomplete folding upon binding mediates Cdk4/cyclin D complex activation by tyrosine phosphorylation of inhibitor p27 protein. J. Biol. Chem..

[31] Blain S.W. (2008). Switching cyclin D-Cdk4 kinase activity on and off. Cell Cycle.

[32] Guiley K.Z., Stevenson J.W., Lou K., Barkovich K.J., Kumarasamy V., Wijeratne T.U., Bunch K.L., Tripathi S., Knudsen E.S., Witkiewicz A.K. (2019). p27 allosterically activates cyclin-dependent kinase 4 and antagonizes palbociclib inhibition. Science.

[33] Aleem E., Kiyokawa H., Kaldis P. (2005). Cdc2-cyclin E complexes regulate the G1/S phase transition. Nat. Cell Biol..

[34] Martin A., Odajima J., Hunt S.L., Dubus P., Ortega S., Malumbres M., Barbacid M. (2005). Cdk2 is dispensable for cell cycle inhibition and tumor suppression mediated by p27(Kip1) and p21(Cip1). Cancer Cell.

[35] Besson A., Hwang H.C., Cicero S., Donovan S.L., Gurian-West M., Johnson D., Clurman B.E., Dyer M.A., Roberts J.M. (2007). Discovery of an oncogenic activity in p27Kip1 that causes stem cell expansion and a multiple tumor phenotype. Genes Dev..

[36] Nakayama K., Ishida N., Shirane M., Inomata A., Inoue T., Shishido N., Horii I., Loh D.Y., Nakayama K. (1996). Mice lacking p27(Kip1) display increased body size, multiple organ hyperplasia, retinal dysplasia, and pituitary tumors. Cell.

[37] Fero M.L., Rivkin M., Tasch M., Porter P., Carow C.E., Firpo E., Polyak K., Tsai L.H., Broudy V., Perlmutter R.M. (1996). A syndrome of multiorgan hyperplasia with features of gigantism, tumorigenesis, and female sterility in p27(Kip1)-deficient mice. Cell.

[38] Kiyokawa H., Kineman R.D., Manova-Todorova K.O., Soares V.C., Hoffman E.S., Ono M., Khanam D., Hayday A.C., Frohman L.A., Koff A. (1996). Enhanced growth of mice lacking the cyclin-dependent kinase inhibitor function of p27(Kip1). Cell.

[39] Bencivenga D., Caldarelli I., Stampone E., Mancini F.P., Balestrieri M.L., Della Ragione F., Borriello A. (2017). p27(Kip1) and human cancers: A reappraisal of a still enigmatic protein. Cancer Lett..

[40] Hnit S.S., Xie C., Yao M., Holst J., Bensoussan A., De Souza P., Li Z., Dong Q. (2015). p27(Kip1) signaling: Transcriptional and post-translational regulation. Int. J. Biochem. Cell Biol..

[41] Montagnoli A., Fiore F., Eytan E., Carrano A.C., Draetta G.F., Hershko A., Pagano M. (1999). Ubiquitination of p27 is regulated by Cdk-dependent phosphorylation and trimeric complex formation. Genes Dev..

[42] Nguyen H., Gitig D.M., Koff A. (1999). Cell-free degradation of p27(kip1), a G1 cyclin-dependent kinase inhibitor, is dependent on CDK2 activity and the proteasome. Mol. Cell. Biol..

[43] Ganoth D., Bornstein G., Ko T.K., Larsen B., Tyers M., Pagano M., Hershko A. (2001). The cell-cycle regulatory protein Cks1 is required for SCF(Skp2)-mediated ubiquitinylation of p27. Nat. Cell Biol..

[44] Pagano M., Tam S.W., Theodoras A.M., Beer-Romero P., Del Sal G., Chau V., Yew P.R., Draetta G.F., Rolfe M. (1995). Role of the ubiquitin-proteasome pathway in regulating abundance of the cyclin-dependent kinase inhibitor p27. Science.

[45] Chu I., Sun J., Arnaout A., Kahn H., Hanna W., Narod S., Sun P., Tan C.K., Hengst L., Slingerland J. (2007). p27 phosphorylation by Src regulates inhibition of cyclin E-Cdk2. Cell.

[46] Chu I.M., Hengst L., Slingerland J.M. (2008). The Cdk inhibitor p27 in human cancer: Prognostic potential and relevance to anticancer therapy. Nat. Rev. Cancer.

[47] Grimmler M., Wang Y., Mund T., Cilensek Z., Keidel E.M., Waddell M.B., Jakel H., Kullmann M., Kriwacki R.W., Hengst L. (2007). Cdk-inhibitory activity and stability of p27Kip1 are directly regulated by oncogenic tyrosine kinases. Cell.

[48] Lacy E.R., Filippov I., Lewis W.S., Otieno S., Xiao L., Weiss S., Hengst L., Kriwacki R.W. (2004). p27 binds cyclin-CDK complexes through a sequential mechanism involving binding-induced protein folding. Nat. Struct. Mol. Biol..

[49] Bencivenga D., Tramontano A., Borgia A., Negri A., Caldarelli I., Oliva A., Perrotta S., Della Ragione F., Borriello A. (2014). P27Kip1 serine 10 phosphorylation determines its metabolism and interaction with cyclin-dependent kinases. Cell Cycle.

[50] Borriello A., Cucciolla V., Criscuolo M., Indaco S., Oliva A., Giovane A., Bencivenga D., Iolascon A., Zappia V., Della Ragione F. (2006). Retinoic acid induces p27Kip1 nuclear accumulation by modulating its phosphorylation. Cancer Res..

[51] Ishida N., Hara T., Kamura T., Yoshida M., Nakayama K., Nakayama K.I. (2002). Phosphorylation of p27Kip1 on serine 10 is required for its binding to CRM1 and nuclear export. J. Biol. Chem..

[52] Connor M.K., Kotchetkov R., Cariou S., Resch A., Lupetti R., Beniston R.G., Melchior F., Hengst L., Slingerland J.M. (2003). CRM1/Ran-mediated nuclear export of p27(Kip1) involves a nuclear export signal and links p27 export and proteolysis. Mol. Biol. Cell.

[53] Kawauchi T., Chihama K., Nabeshima Y., Hoshino M. (2006). Cdk5 phosphorylates and stabilizes p27kip1 contributing to actin organization and cortical neuronal migration. Nat. Cell Biol..

[54] Liang J., Zubovitz J., Petrocelli T., Kotchetkov R., Connor M.K., Han K., Lee J.H., Ciarallo S., Catzavelos C., Beniston R. (2002). PKB/Akt phosphorylates p27, impairs nuclear import of p27 and opposes p27-mediated G1 arrest. Nat. Med..

[55] Shin I., Yakes F.M., Rojo F., Shin N.Y., Bakin A.V., Baselga J., Arteaga C.L. (2002). PKB/Akt mediates cell-cycle progression by phosphorylation of p27(Kip1) at threonine 157 and modulation of its cellular localization. Nat. Med..

[56] Viglietto G., Motti M.L., Bruni P., Melillo R.M., D’Alessio A., Califano D., Vinci F., Chiappetta G., Tsichlis P., Bellacosa A. (2002). Cytoplasmic relocalization and inhibition of the cyclin-dependent kinase inhibitor p27(Kip1) by PKB/Akt-mediated phosphorylation in breast cancer. Nat. Med..

[57] Larrea M.D., Hong F., Wander S.A., da Silva T.G., Helfman D., Lannigan D., Smith J.A., Slingerland J.M. (2009). RSK1 drives p27Kip1 phosphorylation at T198 to promote RhoA inhibition and increase cell motility. Proc. Natl. Acad. Sci. USA.

[58] Liang J., Shao S.H., Xu Z.X., Hennessy B., Ding Z., Larrea M., Kondo S., Dumont D.J., Gutterman J.U., Walker C.L. (2007). The energy sensing LKB1-AMPK pathway regulates p27(kip1) phosphorylation mediating the decision to enter autophagy or apoptosis. Nat. Cell Biol..

[59] Cassimere E.K., Mauvais C., Denicourt C. (2016). p27Kip1 is Required to Mediate a G1 Cell Cycle Arrest Downstream of ATM following Genotoxic Stress. PLoS ONE.

[60] Bachs O., Gallastegui E., Orlando S., Bigas A., Morante-Redolat J.M., Serratosa J., Farinas I., Aligue R., Pujol M.J. (2018). Role of p27(Kip1) as a transcriptional regulator. Oncotarget.

[61] Perez-Luna M., Aguasca M., Perearnau A., Serratosa J., Martinez-Balbas M., Jesus Pujol M., Bachs O. (2012). PCAF regulates the stability of the transcriptional regulator and cyclin-dependent kinase inhibitor p27 Kip1. Nucleic Acids Res..

[62] Pippa R., Espinosa L., Gundem G., Garcia-Escudero R., Dominguez A., Orlando S., Gallastegui E., Saiz C., Besson A., Pujol M.J. (2012). p27Kip1 represses transcription by direct interaction with p130/E2F4 at the promoters of target genes. Oncogene.

[63] Li H., Collado M., Villasante A., Matheu A., Lynch C.J., Canamero M., Rizzoti K., Carneiro C., Martinez G., Vidal A. (2012). p27(Kip1) directly represses Sox2 during embryonic stem cell differentiation. Cell Stem Cell.

[64] Takahashi K., Yamanaka S. (2006). Induction of pluripotent stem cells from mouse embryonic and adult fibroblast cultures by defined factors. Cell.

[65] Yoon H., Kim M., Jang K., Shin M., Besser A., Xiao X., Zhao D., Wander S.A., Briegel K., Morey L. (2019). p27 transcriptionally coregulates cJun to drive programs of tumor progression. Proc. Natl. Acad. Sci. USA.

[66] Cuadrado M., Gutierrez-Martinez P., Swat A., Nebreda A.R., Fernandez-Capetillo O. (2009). p27Kip1 stabilization is essential for the maintenance of cell cycle arrest in response to DNA damage. Cancer Res..

[67] Choi B.K., Fujiwara K., Dayaram T., Darlington Y., Dickerson J., Goodell M.A., Donehower L.A. (2020). WIP1 dephosphorylation of p27(Kip1) Serine 140 destabilizes p27(Kip1) and reverses anti-proliferative effects of ATM phosphorylation. Cell Cycle.

[68] Besson A., Gurian-West M., Schmidt A., Hall A., Roberts J.M. (2004). p27Kip1 modulates cell migration through the regulation of RhoA activation. Genes Dev..

[69] Serres M.P., Kossatz U., Chi Y., Roberts J.M., Malek N.P., Besson A. (2012). p27(Kip1) controls cytokinesis via the regulation of citron kinase activation. J. Clin. Investig..

[70] Baldassarre G., Belletti B., Nicoloso M.S., Schiappacassi M., Vecchione A., Spessotto P., Morrione A., Canzonieri V., Colombatti A. (2005). p27(Kip1)-stathmin interaction influences sarcoma cell migration and invasion. Cancer Cell.

[71] Perchey R.T., Serres M.P., Nowosad A., Creff J., Callot C., Gay A., Manenti S., Margolis R.L., Hatzoglou A., Besson A. (2018). p27(Kip1) regulates the microtubule bundling activity of PRC1. Biochim. Biophys. Acta Mol. Cell Res..

[72] Fabris L., Berton S., Pellizzari I., Segatto I., D’Andrea S., Armenia J., Bomben R., Schiappacassi M., Gattei V., Philips M.R. (2015). p27kip1 controls H-Ras/MAPK activation and cell cycle entry via modulation of MT stability. Proc. Natl. Acad. Sci. USA.

[73] Morelli G., Even A., Gladwyn-Ng I., Le Bail R., Shilian M., Godin J.D., Peyre E., Hassan B.A., Besson A., Rigo J.M. (2018). p27(Kip1) Modulates Axonal Transport by Regulating alpha-Tubulin Acetyltransferase 1 Stability. Cell Rep..

[74] Zhao D., Besser A.H., Wander S.A., Sun J., Zhou W., Wang B., Ince T., Durante M.A., Guo W., Mills G. (2015). Cytoplasmic p27 promotes epithelial-mesenchymal transition and tumor metastasis via STAT3-mediated Twist1 upregulation. Oncogene.

[75] Jeannot P., Nowosad A., Perchey R.T., Callot C., Bennana E., Katsube T., Mayeux P., Guillonneau F., Manenti S., Besson A. (2017). p27(Kip1) promotes invadopodia turnover and invasion through the regulation of the PAK1/Cortactin pathway. Elife.

[76] Gil-Gomez G., Berns A., Brady H.J. (1998). A link between cell cycle and cell death: Bax and Bcl-2 modulate Cdk2 activation during thymocyte apoptosis. EMBO J..

[77] Hiromura K., Pippin J.W., Fero M.L., Roberts J.M., Shankland S.J. (1999). Modulation of apoptosis by the cyclin-dependent kinase inhibitor p27(Kip1). J. Clin. Investig..

[78] Philipp-Staheli J., Payne S.R., Kemp C.J. (2001). p27(Kip1): Regulation and function of a haploinsufficient tumor suppressor and its misregulation in cancer. Exp. Cell Res..

[79] Naruse I., Hoshino H., Dobashi K., Minato K., Saito R., Mori M. (2000). Over-expression of p27kip1 induces growth arrest and apoptosis mediated by changes of pRb expression in lung cancer cell lines. Int. J. Cancer.

[80] Fujieda S., Inuzuka M., Tanaka N., Sunaga H., Fan G.K., Ito T., Sugimoto C., Tsuzuki H., Saito H. (1999). Expression of p27 is associated with Bax expression and spontaneous apoptosis in oral and oropharyngeal carcinoma. Int. J. Cancer.

[81] Hong F., Larrea M.D., Doughty C., Kwiatkowski D.J., Squillace R., Slingerland J.M. (2008). mTOR-raptor binds and activates SGK1 to regulate p27 phosphorylation. Mol. Cell.

[82] White J.P., Billin A.N., Campbell M.E., Russell A.J., Huffman K.M., Kraus W.E. (2018). The AMPK/p27(Kip1) Axis Regulates Autophagy/Apoptosis Decisions in Aged Skeletal Muscle Stem Cells. Stem Cell Rep..

[83] Zada S., Noh H.S., Baek S.M., Ha J.H., Hahm J.R., Kim D.R. (2015). Depletion of p18/LAMTOR1 promotes cell survival via activation of p27(kip1)-dependent autophagy under starvation. Cell Biol. Int..

[84] Medema R.H., Kops G.J., Bos J.L., Burgering B.M. (2000). AFX-like Forkhead transcription factors mediate cell-cycle regulation by Ras and PKB through p27kip1. Nature.

[85] Calnan D.R., Brunet A. (2008). The FoxO code. Oncogene.

[86] Collado M., Medema R.H., Garcia-Cao I., Dubuisson M.L., Barradas M., Glassford J., Rivas C., Burgering B.M., Serrano M., Lam E.W. (2000). Inhibition of the phosphoinositide 3-kinase pathway induces a senescence-like arrest mediated by p27Kip1. J. Biol. Chem..

[87] Bagui T.K., Cui D., Roy S., Mohapatra S., Shor A.C., Ma L., Pledger W.J. (2009). Inhibition of p27Kip1 gene transcription by mitogens. Cell Cycle.

[88] Kullmann M., Gopfert U., Siewe B., Hengst L. (2002). ELAV/Hu proteins inhibit p27 translation via an IRES element in the p27 5′UTR. Genes Dev..

[89] Frenquelli M., Muzio M., Scielzo C., Fazi C., Scarfo L., Rossi C., Ferrari G., Ghia P., Caligaris-Cappio F. (2010). MicroRNA and proliferation control in chronic lymphocytic leukemia: Functional relationship between miR-221/222 cluster and p27. Blood.

[90] Tomoda K., Kubota Y., Arata Y., Mori S., Maeda M., Tanaka T., Yoshida M., Yoneda-Kato N., Kato J.Y. (2002). The cytoplasmic shuttling and subsequent degradation of p27Kip1 mediated by Jab1/CSN5 and the COP9 signalosome complex. J. Biol. Chem..

[91] Patel Y.M., Lane M.D. (2000). Mitotic clonal expansion during preadipocyte differentiation: Calpain-mediated turnover of p27. J. Biol. Chem..

[92] Fagerberg L., Hallstrom B.M., Oksvold P., Kampf C., Djureinovic D., Odeberg J., Habuka M., Tahmasebpoor S., Danielsson A., Edlund K. (2014). Analysis of the human tissue-specific expression by genome-wide integration of transcriptomics and antibody-based proteomics. Mol. Cell. Proteom..

[93] Takahashi K., Nakayama K., Nakayama K. (2000). Mice lacking a CDK inhibitor, p57Kip2, exhibit skeletal abnormalities and growth retardation. J. Biochem..

[94] Susaki E., Nakayama K.I. (2009). Functional similarities and uniqueness of p27 and p57: Insight from a knock-in mouse model. Cell Cycle.

[95] Furutachi S., Matsumoto A., Nakayama K.I., Gotoh Y. (2013). p57 controls adult neural stem cell quiescence and modulates the pace of lifelong neurogenesis. EMBO J..

[96] Matsumoto A., Takeishi S., Kanie T., Susaki E., Onoyama I., Tateishi Y., Nakayama K., Nakayama K.I. (2011). p57 is required for quiescence and maintenance of adult hematopoietic stem cells. Cell Stem Cell.

[97] Nagahama H., Hatakeyama S., Nakayama K., Nagata M., Tomita K., Nakayama K. (2001). Spatial and temporal expression patterns of the cyclin-dependent kinase (CDK) inhibitors p27Kip1 and p57Kip2 during mouse development. Anat. Embryol. (Berl).

[98] Zhang P., Wong C., Liu D., Finegold M., Harper J.W., Elledge S.J. (1999). p21(CIP1) and p57(KIP2) control muscle differentiation at the myogenin step. Genes Dev..

[99] Fahmi M., Ito M. (2019). Evolutionary Approach of Intrinsically Disordered CIP/KIP Proteins. Sci. Rep..

[100] Kamura T., Hara T., Kotoshiba S., Yada M., Ishida N., Imaki H., Hatakeyama S., Nakayama K., Nakayama K.I. (2003). Degradation of p57Kip2 mediated by SCFSkp2-dependent ubiquitylation. Proc. Natl. Acad. Sci. USA.

[101] Joseph B., Andersson E.R., Vlachos P., Sodersten E., Liu L., Teixeira A.I., Hermanson O. (2009). p57Kip2 is a repressor of Mash1 activity and neuronal differentiation in neural stem cells. Cell Death Differ..

[102] Joseph B., Wallen-Mackenzie A., Benoit G., Murata T., Joodmardi E., Okret S., Perlmann T. (2003). p57(Kip2) cooperates with Nurr1 in developing dopamine cells. Proc. Natl. Acad. Sci. USA.

[103] Joaquin M., Gubern A., Gonzalez-Nunez D., Josue Ruiz E., Ferreiro I., de Nadal E., Nebreda A.R., Posas F. (2012). The p57 CDKi integrates stress signals into cell-cycle progression to promote cell survival upon stress. EMBO J..

[104] Thayer M.J., Tapscott S.J., Davis R.L., Wright W.E., Lassar A.B., Weintraub H. (1989). Positive autoregulation of the myogenic determination gene MyoD1. Cell.

[105] Reynaud E.G., Pelpel K., Guillier M., Leibovitch M.P., Leibovitch S.A. (1999). p57(Kip2) stabilizes the MyoD protein by inhibiting cyclin E-Cdk2 kinase activity in growing myoblasts. Mol. Cell. Biol..

[106] Yokoo T., Toyoshima H., Miura M., Wang Y., Iida K.T., Suzuki H., Sone H., Shimano H., Gotoda T., Nishimori S. (2003). p57Kip2 regulates actin dynamics by binding and translocating LIM-kinase 1 to the nucleus. J. Biol. Chem..

[107] Vlachos P., Joseph B. (2009). The Cdk inhibitor p57(Kip2) controls LIM-kinase 1 activity and regulates actin cytoskeleton dynamics. Oncogene.

[108] Itoh Y., Masuyama N., Nakayama K., Nakayama K.I., Gotoh Y. (2007). The cyclin-dependent kinase inhibitors p57 and p27 regulate neuronal migration in the developing mouse neocortex. J. Biol. Chem..

[109] Arboleda V.A., Lee H., Parnaik R., Fleming A., Banerjee A., Ferraz-de-Souza B., Delot E.C., Rodriguez-Fernandez I.A., Braslavsky D., Bergada I. (2012). Mutations in the PCNA-binding domain of CDKN1C cause IMAGe syndrome. Nat. Genet..

[110] Davis R.J. (2000). Signal transduction by the JNK group of MAP kinases. Cell.

[111] Kavanagh K.T., Hafer L.J., Kim D.W., Mann K.K., Sherr D.H., Rogers A.E., Sonenshein G.E. (2001). Green tea extracts decrease carcinogen-induced mammary tumor burden in rats and rate of breast cancer cell proliferation in culture. J. Cell. Biochem..

[112] Bulavin D.V., Higashimoto Y., Popoff I.J., Gaarde W.A., Basrur V., Potapova O., Appella E., Fornace A.J. (2001). Initiation of a G2/M checkpoint after ultraviolet radiation requires p38 kinase. Nature.

[113] Samuelsson M.K., Pazirandeh A., Okret S. (2002). A pro-apoptotic effect of the CDK inhibitor p57(Kip2) on staurosporine-induced apoptosis in HeLa cells. Biochem. Biophys. Res. Commun..

[114] Jia H., Cong Q., Chua J.F., Liu H., Xia X., Zhang X., Lin J., Habib S.L., Ao J., Zuo Q. (2015). p57Kip2 is an unrecognized DNA damage response effector molecule that functions in tumor suppression and chemoresistance. Oncogene.

[115] Jiang Y., Lo W., Akhmametyeva E.M., Chang L.S. (2006). Over-expression of p73beta results in apoptotic death of post-mitotic hNT neurons. J. Neurol. Sci..

[116] Gonzalez S., Perez-Perez M.M., Hernando E., Serrano M., Cordon-Cardo C. (2005). p73beta-Mediated apoptosis requires p57kip2 induction and IEX-1 inhibition. Cancer Res..

[117] Stampone E., Bencivenga D., Barone C., Aulitto A., Verace F., Della Ragione F., Borriello A. (2020). High Dosage Lithium Treatment Induces DNA Damage and p57(Kip2) Decrease. Int. J. Mol. Sci..

[118] Li W.Y., Li Q., Jing L., Wu T., Han L.L., Wang Y., Yu S.Z., Nan K.J., Guo H. (2019). P57-mediated autophagy promotes the efficacy of EGFR inhibitors in hepatocellular carcinoma. Liver Int..

[119] Schwarze S.R., Shi Y., Fu V.X., Watson P.A., Jarrard D.F. (2001). Role of cyclin-dependent kinase inhibitors in the growth arrest at senescence in human prostate epithelial and uroepithelial cells. Oncogene.

[120] Giovannini C., Gramantieri L., Minguzzi M., Fornari F., Chieco P., Grazi G.L., Bolondi L. (2012). CDKN1C/P57 is regulated by the Notch target gene Hes1 and induces senescence in human hepatocellular carcinoma. Am. J. Pathol..

[121] Tsugu A., Sakai K., Dirks P.B., Jung S., Weksberg R., Fei Y.L., Mondal S., Ivanchuk S., Ackerley C., Hamel P.A. (2000). Expression of p57(KIP2) potently blocks the growth of human astrocytomas and induces cell senescence. Am. J. Pathol..

[122] Velicky P., Meinhardt G., Plessl K., Vondra S., Weiss T., Haslinger P., Lendl T., Aumayr K., Mairhofer M., Zhu X. (2018). Genome amplification and cellular senescence are hallmarks of human placenta development. PLoS Genet..

[123] Miglionico R., Ostuni A., Armentano M.F., Milella L., Crescenzi E., Carmosino M., Bisaccia F. (2017). ABCC6 knockdown in HepG2 cells induces a senescent-like cell phenotype. Cell. Mol. Biol. Lett..

[124] Stampone E., Caldarelli I., Zullo A., Bencivenga D., Mancini F.P., Della Ragione F., Borriello A. (2018). Genetic and Epigenetic Control of CDKN1C Expression: Importance in Cell Commitment and Differentiation, Tissue Homeostasis and Human Diseases. Int. J. Mol. Sci..

[125] Andresini O., Ciotti A., Rossi M.N., Battistelli C., Carbone M., Maione R. (2016). A cross-talk between DNA methylation and H3 lysine 9 dimethylation at the KvDMR1 region controls the induction of Cdkn1c in muscle cells. Epigenetics.

[126] John R.M., Lefebvre L. (2011). Developmental regulation of somatic imprints. Differentiation.

[127] Bhogal B., Arnaudo A., Dymkowski A., Best A., Davis T.L. (2004). Methylation at mouse Cdkn1c is acquired during postimplantation development and functions to maintain imprinted expression. Genomics.

[128] Busanello A., Battistelli C., Carbone M., Mostocotto C., Maione R. (2012). MyoD regulates p57kip2 expression by interacting with a distant cis-element and modifying a higher order chromatin structure. J. Clin. Investig..

[129] El Kharroubi A., Piras G., Stewart C.L. (2001). DNA demethylation reactivates a subset of imprinted genes in uniparental mouse embryonic fibroblasts. J. Biol. Chem..

[130] Algar E.M., Muscat A., Dagar V., Rickert C., Chow C.W., Biegel J.A., Ekert P.G., Saffery R., Craig J., Johnstone R.W. (2009). Imprinted CDKN1C is a tumor suppressor in rhabdoid tumor and activated by restoration of SMARCB1 and histone deacetylase inhibitors. PLoS ONE.

[131] Cucciolla V., Borriello A., Criscuolo M., Sinisi A.A., Bencivenga D., Tramontano A., Scudieri A.C., Oliva A., Zappia V., Della Ragione F. (2008). Histone deacetylase inhibitors upregulate p57Kip2 level by enhancing its expression through Sp1 transcription factor. Carcinogenesis.

[132] Maiso P., Carvajal-Vergara X., Ocio E.M., Lopez-Perez R., Mateo G., Gutierrez N., Atadja P., Pandiella A., San Miguel J.F. (2006). The histone deacetylase inhibitor LBH589 is a potent antimyeloma agent that overcomes drug resistance. Cancer Res..

[133] Dauphinot L., De Oliveira C., Melot T., Sevenet N., Thomas V., Weissman B.E., Delattre O. (2001). Analysis of the expression of cell cycle regulators in Ewing cell lines: EWS-FLI-1 modulates p57KIP2and c-Myc expression. Oncogene.

[134] Tokino T., Urano T., Furuhata T., Matsushima M., Miyatsu T., Sasaki S., Nakamura Y. (1996). Characterization of the human p57KIP2 gene: Alternative splicing, insertion/deletion polymorphisms in VNTR sequences in the coding region, and mutational analysis. Hum. Genet..

[135] Figliola R., Busanello A., Vaccarello G., Maione R. (2008). Regulation of p57(KIP2) during muscle differentiation: Role of Egr1, Sp1 and DNA hypomethylation. J. Mol. Biol..

[136] Svaren J., Ehrig T., Abdulkadir S.A., Ehrengruber M.U., Watson M.A., Milbrandt J. (2000). EGR1 target genes in prostate carcinoma cells identified by microarray analysis. J. Biol. Chem..

[137] Beretta C., Chiarelli A., Testoni B., Mantovani R., Guerrini L. (2005). Regulation of the cyclin-dependent kinase inhibitor p57Kip2 expression by p63. Cell Cycle.

[138] Balint E., Phillips A.C., Kozlov S., Stewart C.L., Vousden K.H. (2002). Induction of p57(KIP2) expression by p73beta. Proc. Natl. Acad. Sci. USA.

[139] Alheim K., Corness J., Samuelsson M.K., Bladh L.G., Murata T., Nilsson T., Okret S. (2003). Identification of a functional glucocorticoid response element in the promoter of the cyclin-dependent kinase inhibitor p57Kip2. J. Mol. Endocrinol..

[140] Samuelsson M.K., Pazirandeh A., Davani B., Okret S. (1999). p57Kip2, a glucocorticoid-induced inhibitor of cell cycle progression in HeLa cells. Mol. Endocrinol..

[141] Medina R., Zaidi S.K., Liu C.G., Stein J.L., van Wijnen A.J., Croce C.M., Stein G.S. (2008). MicroRNAs 221 and 222 bypass quiescence and compromise cell survival. Cancer Res..

[142] Kim Y.K., Yu J., Han T.S., Park S.Y., Namkoong B., Kim D.H., Hur K., Yoo M.W., Lee H.J., Yang H.K. (2009). Functional links between clustered microRNAs: Suppression of cell-cycle inhibitors by microRNA clusters in gastric cancer. Nucleic Acids Res..

[143] Lopez-Nieva P., Fernandez-Navarro P., Vaquero-Lorenzo C., Villa-Morales M., Grana-Castro O., Cobos-Fernandez M.A., Lopez-Lorenzo J.L., Llamas P., Gonzalez-Sanchez L., Sastre I. (2018). RNA-Seq reveals the existence of a CDKN1C-E2F1-TP53 axis that is altered in human T-cell lymphoblastic lymphomas. BMC Cancer.

[144] Wang J., Zhao H., Yu J., Xu X., Liu W., Jing H., Li N., Tang Y., Li Y., Cai J. (2019). MiR-92b targets p57kip2 to modulate the resistance of hepatocellular carcinoma (HCC) to ionizing radiation (IR)-based radiotherapy. Biomed. Pharmacother..

[145] Jang M., Cai L., Udeani G.O., Slowing K.V., Thomas C.F., Beecher C.W., Fong H.H., Farnsworth N.R., Kinghorn A.D., Mehta R.G. (1997). Cancer chemopreventive activity of resveratrol, a natural product derived from grapes. Science.

[146] Russo G.L., Tedesco I., Spagnuolo C., Russo M. (2017). Antioxidant polyphenols in cancer treatment: Friend, foe or foil?. Semin. Cancer Biol..

[147] Ahmad N., Cheng P., Mukhtar H. (2000). Cell cycle dysregulation by green tea polyphenol epigallocatechin-3-gallate. Biochem. Biophys. Res. Commun..

[148] Gupta S., Hussain T., Mukhtar H. (2003). Molecular pathway for (-)-epigallocatechin-3-gallate-induced cell cycle arrest and apoptosis of human prostate carcinoma cells. Arch. Biochem. Biophys..

[149] Sah J.F., Balasubramanian S., Eckert R.L., Rorke E.A. (2004). Epigallocatechin-3-gallate inhibits epidermal growth factor receptor signaling pathway. Evidence for direct inhibition of ERK1/2 and AKT kinases. J. Biol. Chem..

[150] Kim C.G., Lee H., Gupta N., Ramachandran S., Kaushik I., Srivastava S., Kim S.H., Srivastava S.K. (2018). Role of Forkhead Box Class O proteins in cancer progression and metastasis. Semin. Cancer Biol..

[151] Eddy S.F., Kane S.E., Sonenshein G.E. (2007). Trastuzumab-resistant HER2-driven breast cancer cells are sensitive to epigallocatechin-3 gallate. Cancer Res..

[152] Nam S., Smith D.M., Dou Q.P. (2001). Ester bond-containing tea polyphenols potently inhibit proteasome activity in vitro and in vivo. J. Biol. Chem..

[153] Borriello A., Bencivenga D., Caldarelli I., Tramontano A., Borgia A., Pirozzi A.V., Oliva A., Della Ragione F. (2013). Resveratrol and cancer treatment: Is hormesis a yet unsolved matter?. Curr. Pharm. Des..

[154] Kuwajerwala N., Cifuentes E., Gautam S., Menon M., Barrack E.R., Reddy G.P. (2002). Resveratrol induces prostate cancer cell entry into s phase and inhibits DNA synthesis. Cancer Res..

[155] Benitez D.A., Pozo-Guisado E., Alvarez-Barrientos A., Fernandez-Salguero P.M., Castellon E.A. (2007). Mechanisms involved in resveratrol-induced apoptosis and cell cycle arrest in prostate cancer-derived cell lines. J. Androl..

[156] Wang T.T., Schoene N.W., Kim Y.S., Mizuno C.S., Rimando A.M. (2010). Differential effects of resveratrol and its naturally occurring methylether analogs on cell cycle and apoptosis in human androgen-responsive LNCaP cancer cells. Mol. Nutr. Food Res..

[157] Chen Q., Ganapathy S., Singh K.P., Shankar S., Srivastava R.K. (2010). Resveratrol induces growth arrest and apoptosis through activation of FOXO transcription factors in prostate cancer cells. PLoS ONE.

[158] Ganapathy S., Chen Q., Singh K.P., Shankar S., Srivastava R.K. (2010). Resveratrol enhances antitumor activity of TRAIL in prostate cancer xenografts through activation of FOXO transcription factor. PLoS ONE.

[159] Roy S.K., Chen Q., Fu J., Shankar S., Srivastava R.K. (2011). Resveratrol inhibits growth of orthotopic pancreatic tumors through activation of FOXO transcription factors. PLoS ONE.

[160] Niu X.F., Liu B.Q., Du Z.X., Gao Y.Y., Li C., Li N., Guan Y., Wang H.Q. (2011). Resveratrol protects leukemic cells against cytotoxicity induced by proteasome inhibitors via induction of FOXO1 and p27Kip1. BMC Cancer.

[161] Vanamala J., Reddivari L., Radhakrishnan S., Tarver C. (2010). Resveratrol suppresses IGF-1 induced human colon cancer cell proliferation and elevates apoptosis via suppression of IGF-1R/Wnt and activation of p53 signaling pathways. BMC Cancer.

[162] Lee K.A., Lee Y.J., Ban J.O., Lee Y.J., Lee S.H., Cho M.K., Nam H.S., Hong J.T., Shim J.H. (2012). The flavonoid resveratrol suppresses growth of human malignant pleural mesothelioma cells through direct inhibition of specificity protein 1. Int. J. Mol. Med..

[163] Wolter F., Stein J. (2002). Resveratrol enhances the differentiation induced by butyrate in caco-2 colon cancer cells. J. Nutr..

[164] Kubota T., Uemura Y., Kobayashi M., Taguchi H. (2003). Combined effects of resveratrol and paclitaxel on lung cancer cells. Anticancer Res..

[165] Singh S.K., Banerjee S., Acosta E.P., Lillard J.W., Singh R. (2017). Resveratrol induces cell cycle arrest and apoptosis with docetaxel in prostate cancer cells via a p53/p21WAF1/CIP1 and p27KIP1 pathway. Oncotarget.

[166] Haider U.G., Sorescu D., Griendling K.K., Vollmar A.M., Dirsch V.M. (2003). Resveratrol increases serine15-phosphorylated but transcriptionally impaired p53 and induces a reversible DNA replication block in serum-activated vascular smooth muscle cells. Mol. Pharmacol..

[167] Lee B., Moon S.K. (2005). Resveratrol inhibits TNF-alpha-induced proliferation and matrix metalloproteinase expression in human vascular smooth muscle cells. J. Nutr..

[168] Nam S., Smith D.M., Dou Q.P. (2001). Tannic acid potently inhibits tumor cell proteasome activity, increases p27 and Bax expression, and induces G1 arrest and apoptosis. Cancer Epidemiol. Prev. Biomark..

[169] Shishodia S., Sethi G., Ahn K.S., Aggarwal B.B. (2007). Guggulsterone inhibits tumor cell proliferation, induces S-phase arrest, and promotes apoptosis through activation of c-Jun N-terminal kinase, suppression of Akt pathway, and downregulation of antiapoptotic gene products. Biochem. Pharmacol..

[170] Lee K.W., Kim H.J., Lee Y.S., Park H.J., Choi J.W., Ha J., Lee K.T. (2007). Acteoside inhibits human promyelocytic HL-60 leukemia cell proliferation via inducing cell cycle arrest at G0/G1 phase and differentiation into monocyte. Carcinogenesis.

[171] Steiner M., Priel I., Giat J., Levy J., Sharoni Y., Danilenko M. (2001). Carnosic acid inhibits proliferation and augments differentiation of human leukemic cells induced by 1,25-dihydroxyvitamin D3 and retinoic acid. Nutr. Cancer.

[172] Al Dhaheri Y., Attoub S., Ramadan G., Arafat K., Bajbouj K., Karuvantevida N., AbuQamar S., Eid A., Iratni R. (2014). Carnosol induces ROS-mediated beclin1-independent autophagy and apoptosis in triple negative breast cancer. PLoS ONE.

[173] Gao Y., Yin J., Tu Y., Chen Y.C. (2019). Theaflavin-3,3′-Digallate Suppresses Human Ovarian Carcinoma OVCAR-3 Cells by Regulating the Checkpoint Kinase 2 and p27 kip1 Pathways. Molecules.

[174] Terzuoli E., Nannelli G., Frosini M., Giachetti A., Ziche M., Donnini S. (2017). Inhibition of cell cycle progression by the hydroxytyrosol-cetuximab combination yields enhanced chemotherapeutic efficacy in colon cancer cells. Oncotarget.

[175] Della Ragione F., Cucciolla V., Criniti V., Indaco S., Borriello A., Zappia V. (2002). Antioxidants induce different phenotypes by a distinct modulation of signal transduction. FEBS Lett..

[176] Sankaranarayanan R., Valiveti C.K., Kumar D.R., Van Slambrouck S., Kesharwani S.S., Seefeldt T., Scaria J., Tummala H., Bhat G.J. (2019). The Flavonoid Metabolite 2, 4, 6-Trihydroxybenzoic Acid Is a CDK Inhibitor and an Anti-Proliferative Agent: A Potential Role in Cancer Prevention. Cancers (Basel).

[177] Hsu S., Lewis J.B., Borke J.L., Singh B., Dickinson D.P., Caughman G.B., Athar M., Drake L., Aiken A.C., Huynh C.T. (2001). Chemopreventive effects of green tea polyphenols correlate with reversible induction of p57 expression. Anticancer Res..

[178] Hsu S., Yu F.S., Lewis J., Singh B., Borke J., Osaki T., Athar M., Schuster G. (2002). Induction of p57 is required for cell survival when exposed to green tea polyphenols. Anticancer Res..

[179] Hsu S., Bollag W.B., Lewis J., Huang Q., Singh B., Sharawy M., Yamamoto T., Schuster G. (2003). Green tea polyphenols induce differentiation and proliferation in epidermal keratinocytes. J. Pharmacol. Exp. Ther..

[180] Hsu S.D., Singh B.B., Lewis J.B., Borke J.L., Dickinson D.P., Drake L., Caughman G.B., Schuster G.S. (2002). Chemoprevention of oral cancer by green tea. Gen Dent.

[181] Hsu S., Yamamoto T., Borke J., Walsh D.S., Singh B., Rao S., Takaaki K., Nah-Do N., Lapp C., Lapp D. (2005). Green tea polyphenol-induced epidermal keratinocyte differentiation is associated with coordinated expression of p57/KIP2 and caspase 14. J Pharmacol Exp Ther.

[182] Balasubramanian S., Efimova T., Eckert R.L. (2002). Green tea polyphenol stimulates a Ras, MEKK1, MEK3, and p38 cascade to increase activator protein 1 factor-dependent involucrin gene expression in normal human keratinocytes. J. Biol. Chem..

[183] Russo G.L., Spagnuolo C., Russo M., Tedesco I., Moccia S., Cervellera C. (2020). Mechanisms of aging and potential role of selected polyphenols in extending healthspan. Biochem. Pharmacol..

[184] Chou C.C., Yang J.S., Lu H.F., Ip S.W., Lo C., Wu C.C., Lin J.P., Tang N.Y., Chung J.G., Chou M.J. (2010). Quercetin-mediated cell cycle arrest and apoptosis involving activation of a caspase cascade through the mitochondrial pathway in human breast cancer MCF-7 cells. Arch. Pharm. Res..

[185] Kopustinskiene D.M., Jakstas V., Savickas A., Bernatoniene J. (2020). Flavonoids as Anticancer Agents. Nutrients.

